# Sunlight‐Driven Green Synthesis of Platinum Nanoparticles From Double‐Vacancy Halide Perovskite Precursors for Methanol Reforming

**DOI:** 10.1002/chem.202503192

**Published:** 2026-05-11

**Authors:** Kevin Mego, Saray Chacón‐Ruiz, Pedro Ruiz‐Campos, Herme G. Baldoví, Pedro Atienzar

**Affiliations:** ^1^ Instituto de Tecnología Química (ITQ) Consejo Superior de Investigaciones Científicas‐Universitat Politècnica de València Valencia Spain; ^2^ Universidad Científica del Sur Lima Perú; ^3^ Departamento de Química Universitat Politècnica de València Valencia Spain

**Keywords:** green synthesis, lead‐free perovskites, methanol reforming reaction, platinum nanoparticles

## Abstract

The development of synthetic procedures for preparing metal nanoparticles with controlled size using sunlight opens new avenues toward more sustainable methods with lower energy consumption. Herein, we propose the use of double‐vacancy halide perovskites as precursors for engineering highly crystalline and small platinum nanoparticles (< 3 nm) under simulated sunlight irradiation, using low concentration of methanol as sacrificial agent at room temperature. The formation of Pt NP was dependent on the methanol concentration impacting in the final Pt particle size distribution and time formation. After purification, this narrow sized Pt NP were employed for the methanol reforming reaction, showing excellent activity after 4 h and high selectivity toward hydrogen production (4.18 mmol·h^−1^ of H_2_), along with other carbon products such as CO (2.26 mmol·h^−1^), CH_4_ (0.54 mmol·h^−1^), CO_2_ (0.28 mmol·h^−1^), and C_2_ species (0.17 mmol·h^−1^).

## Graphical abstract

Sunlight‐driven synthesis offers a green and energy‐efficient route to metallic nanoparticles. Here, double‐vacancy halide perovskites (Cs_2_PtX_6_, X = Cl, Br) act as photoactive precursors for the in‐situ formation of ultrasmall (< 3 nm) platinum nanoparticles under simulated sunlight at room temperature. The resulting Pt nanocatalyst exhibit high crystallinity and outstanding activity in the methanol reforming reaction, highlighting a sustainable strategy for solar‐to‐chemical energy conversion.

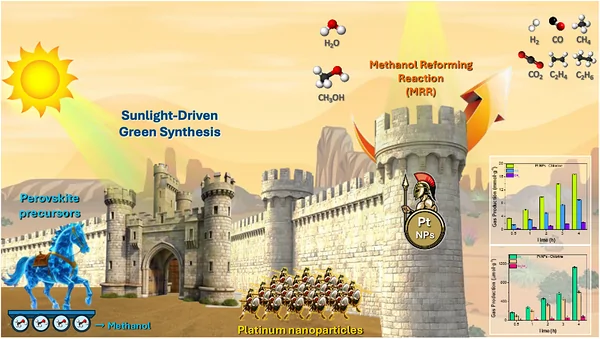

## 1 Introduction

Platinum nanoparticles (Pt NPs) are among the most studied and technologically significant nanomaterials due to their exceptional physicochemical properties, including high electrical conductivity, excellent thermal stability, and remarkable catalytic activity [1]. These features have positioned Pt NPs as key components in diverse applications ranging from biomedical devices and fuel cells to electrochemical sensors and environmental remediation systems [2, 3, 4, 5]. Of particular relevance is their catalytic performance in hydrogenation reactions and reforming processes, where Pt‐based catalysts play a crucial role in converting small organic molecules into value‐added products [6, 7, 8, 9]. In this context, the catalytic reforming of methanol has garnered increasing attention as a pathway for producing sustainable energy‐relevant subproducts such as hydrogen (H_2_), carbon monoxide (CO), methane (CH_4_), ethylene (C_2_H_4_), ethane (C_2_H_6_), and carbon dioxide (CO_2_).

Over the past decades, various synthetic strategies have been developed for the preparation of Pt NPs, broadly categorized into physical, chemical, and biological methods. Physical techniques such as laser ablation, thermal evaporation, and sputtering typically yield well‐defined NPs but require high energy input, costly instrumentation, and often suffer from limited scalability [10]. Chemical approaches, including the widely employed polyol method [11], chemical reduction in aqueous or organic solvents [12, 13], and microwave‐assisted synthesis [14], have enabled better control over particle morphology, size distribution, and surface functionalities. However, many of these routes rely on harsh reaction conditions, toxic reducing agents (e.g., sodium borohydride, hydrazine), and stabilizing polymers that may interfere with catalytic performance. In contrast, biologically mediated methods‐employing plant extracts, microbial agents, or biopolymers‐represent a more sustainable alternative, enabling Pt NP synthesis under milder conditions using benign reductants such as flavonoids or ascorbic acid [15, 16, 17, 18]. Nonetheless, challenges remain in achieving monodispersity, high crystallinity, and reproducibility at scale. A comparative overview of representative synthetic methods for Pt NPs, including physical, chemical, and green approaches, is summarized in Table S1.

Among the recent advances, sunlight‐driven NP synthesis has emerged as an attractive green alternative to conventional methods. This approach harnesses the energy of light, either from natural sunlight or simulated solar sources, to induce reduction of metal precursors in solution, often in the presence of benign additives such as alcohols [19]. The sunlight‐driven route offers several notable advantages: it significantly reduces reaction times, avoids the need for external heating, and enables the formation of pure, crystalline, and monodisperse NPs with enhanced stability in aqueous media. Furthermore, the ambient reaction conditions and the absence of toxic reagents make it a highly appealing strategy in the context of sustainable nanomaterials synthesis, aligning with the principles of green chemistry.

Despite these promising developments, to date, there are no reports of sunlight‐assisted synthesis of Pt NPs from halide double‐vacancy halide perovskite precursors, which could provide a novel platform for both efficient NP formation and catalytic performance. Halide perovskites of the type of Cs_2_PtX_6_ (*X* = Cl, Br), characterized by their vacant B‐site octahedral structure, present an intriguing opportunity as platinum‐rich, structurally tunable precursors for NP synthesis. Their sunlight simulated responsiveness and aqueous solubility make them especially well‐suited for light‐mediated processes.

The reduction of the B‐site metal in perovskites has generally been considered as a light‐induced degradation pathway [20, 21]. Recent studies have also investigated the nucleation of silver NPs in silver‐based double‐perovskite nanocrystals under light irradiation [22]. However, no studies have considered this perovskite degradation process as a potential strategy to produce metal NPs with tunable composition, size, and crystallinity.

Therefore, in this study, we present the first comprehensive investigation into the green sunlight‐driven synthesis of Pt NPs from double‐vacancy perovskite precursors Cs_2_PtCl_6_ and Cs_2_PtBr_6_, using simulated sunlight irradiation in an aqueous‐methanol medium under argon atmosphere to transform the octahedrally coordinated platinum cation in metal NPs. This sunlight‐driven method yields monodisperse Pt NPs with high crystallinity and colloidal stability, without requiring any additional surfactants or harsh reducing agents. Furthermore, we demonstrate that the resulting NPs exhibit significant catalytic activity in methanol reforming reactions (MRRs), facilitating the transformation of methanol into a suite of energetically relevant subproducts including H_2_, CO, CH_4_, C_2_H_4_, C_2_H_6_, and CO_2_ [23, 24, 25, 26]. This work not only introduces a novel, sustainable synthetic route for platinum nanomaterials using double‐vacancy perovskite precursors, but also reveals the untapped catalytic potential of perovskite‐derived Pt NPs in the context of valorization of biomass alcohols to other chemicals of interest. Moreover, this synthetic route opens new avenues for the preparation of other metal NPs by tuning the octahedrally coordinated metal cation within the perovskite structure.

## 2 Results and Discussion

Double‐vacancy halide perovskites were synthesized hydrothermally following a procedure published by Mego et al. (see Experimental Section) [27]. Consistent with this approach, Pt NPs were synthesized from double‐vacancy halide perovskites, Cs_2_PtCl_6_ and Cs_2_PtBr_6_, as platinum‐rich precursors. The synthetic route described herein highlights the fundamental role of simulated sunlight irradiation, in synergy with methanol as an electron donor, in driving the photoreduction of the perovskite precursors in an aqueous medium. This process capitalizes on the dual function of methanol: first, as an electron donor under photoexcitation, enabling the efficient reduction of Pt^4+^ to metallic Pt^0^; and second, as a benign additive that stabilizes NPs during growth and prevents uncontrolled aggregation. The entire procedure is conducted under an inert argon atmosphere to suppress oxidative side reactions, ensuring that the resulting Pt NPs retain high purity, crystalline structure, small particle size, and well‐defined structural features. After Pt NP formation, NP were purified by washing samples with centrifugation, therefore ensuring that cesium and halides precursors were removed before using Pt NP in catalysis.

In addition to their synthesis, the catalytic performance of the freshly prepared Pt NPs was evaluated in the MRR. This process was chosen not only for its relevance in sustainable energy conversion but also because it provides a stringent benchmark for catalytic activity, selectivity, and stability under operational conditions.

In addition to their synthesis, the catalytic performance of the freshly prepared Pt NPs was evaluated in the MRR. This process was chosen for its relevance in sustainable energy conversion and because it provides a stringent benchmark for catalytic activity, selectivity, and stability under operational conditions.

**FIGURE 1:**
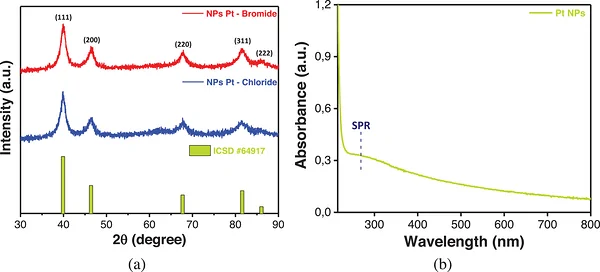
(a) x‐Ray diffraction (XRD) patterns of Pt NPs‐synthesized from Cs_2_PtCl_6_ (blue solid line), Cs_2_PtBr_6_ (red solid line), and Pt NPs control reference ICSD code: (#64917) (green solid line). (b) UV–vis absorption spectra of Pt NPs synthesized.

The crystallinity and phase purity of the synthesized NPs were confirmed by powder x‐ray diffraction (XRD) analysis (Figure 1a). The diffraction patterns obtained for both Cs_2_PtCl_6_ and Cs_2_PtBr_6_‐derived Pt NPs exhibited well‐defined peaks that closely match the standard face‐centered cubic (fcc) structure of metallic platinum, in excellent agreement with the seminal data reported by Barth and Lunde [28]. No additional peaks attributable to unreacted precursors or secondary phases were observed. Consistent with the XRD‐confirmed fcc Pt phase, the UV–vis spectrum (Figure 1b) shows a characteristic Pt NP strong surface plasma resonance (SPR) peak centered at 268 nm, deriving from collective oscillation of conduction electrons strongly damped in Pt [29, 30]. The absence of additional absorption features supports complete precursor conversion and corroborates the formation of highly crystalline, phase‐pure Pt NPs.

Importantly, no discernible shifts or changes in the 2*θ* positions were detected, indicating that the lattice parameters of the Pt NPs were consistent with those reported in the ICSD data file #64917 [28], which relies on the crystalline nature of the materials. Moreover, the diffraction peaks are sharp, with broadening limited to that expected from finite crystallite size, supporting the high crystallinity of the NPs obtained via simulated sunlight irradiation.

This finding is noteworthy when compared with Pt NPs synthesized via alternative green and non‐green methods, such as sonochemical [31], ultrasonic [32], or conventional thermal reduction [33] routes, which often yield products with broader diffraction peaks, indicative of lower crystallinity or a higher density of defects.

**FIGURE 2:**
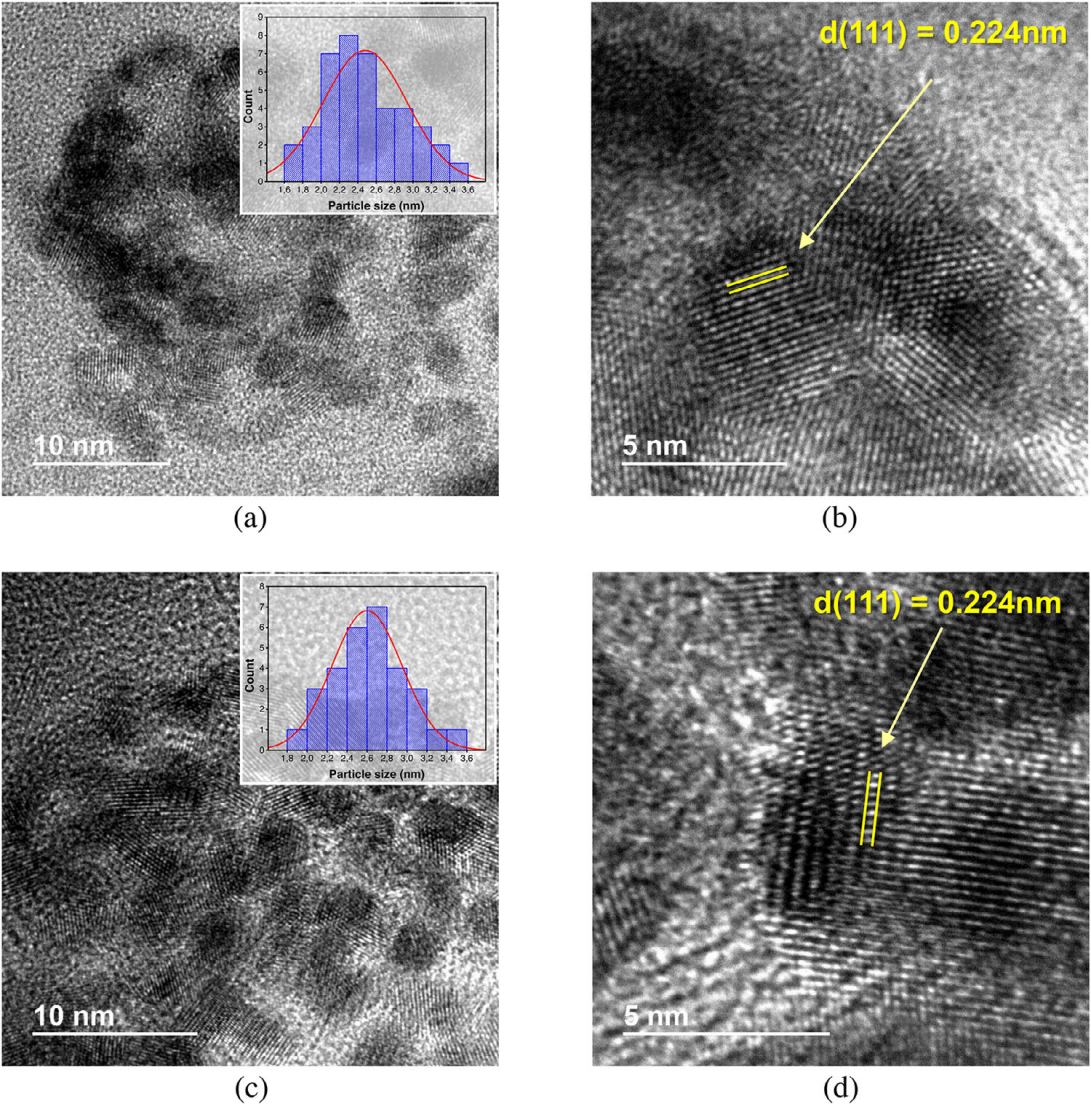
Structural and morphological characterization of Pt nanoparticles (NPs) synthesized from halide perovskite precursors. HR‐TEM images of Pt NPs prepared from (a, b) Cs_2_PtCl_6_ and (c, d) Cs_2_PtBr_6_, (a, c) show an overview image with the corresponding average particle size and distribution histograms of Pt NPs provided as right insets. Panels (b, d) are high‐resolution images revealing lattice fringes with interplanar spacings of *d* = 0.224 nm, consistent with the (111) crystallographic planes of Pt NPs.

To enable a meaningful comparison of catalytic performance between Pt NPs derived from the two perovskite precursors, the synthesis parameters were optimized to produce NPs with a narrow size distribution of 2‐3 nm (Figure 2). Moreover, the decomposition rate of the perovskite into Pt NPs is closely related to the solubility of Cs in alcoholic solutions compared with water. At very low methanol concentrations (0.4 mL MeOH in 10 mL Milli‐Q water), the perovskite decomposition is almost complete in the same reaction time, resulting in uniformly small Pt NPs (∼2 nm). However, upon addition of 1.6 mL MeOH, Pt NPs with a size of 4.3–5.4 nm were observed (Figures S1 and S3). Likewise, when adding 2.8 mL MeOH, larger Pt NPs (6.5–6.8 nm) with a broader size distribution are formed, along with detectable remnants of the initial perovskite as large crystals, as shown in Figures S2 and S4. This suggests that methanol concentration modulates the rate of perovskite decomposition, thereby influencing the final size and distribution of Pt NPs. As methanol concentration increases, it alters the solubility and diffusion properties of the precursor, thereby influencing NP formation kinetics. The higher concentration of methanol stabilizes the perovskite phase and retards the decomposition rate, resulting in a bigger NP size. This trend is consistent with the markedly different solubility of Cs‐containing phases in alcohol‐rich media: Cs_2_PtCl_6_ has been reported to be insoluble in methanol, favoring precipitation/persistence of Cs–Pt halide phases [34]. This finding is clearly reflected in Table S2, which summarizes the size distributions for both Pt NPs‐chloride and Pt NPs‐bromide at different methanol concentrations.

This size regime is particularly relevant for catalysis, as it ensures a high surface‐to‐volume ratio and maximizes the number of active sites available for reaction. HR‐TEM analysis confirmed the high crystallinity of the synthesized Pt NPs, as evidenced by the well‐defined lattice fringes with an interplanar spacing of 0.224 nm, which is characteristic of the metallic Pt (111) planes [35]. This atomic‐level structural confirmation is crucial, as it verifies the formation of phase‐pure, crystalline Pt, which is directly linked to enhanced catalytic activity and stability.

Energy dispersive x‐ray analysis (EDX) study confirmed the elemental platinum in the prepared NPs. The elemental and weight percentages of the Pt NPs synthesized by both precursors are presented in Figure S5a,b and Table S3. The EDX data performed from the precursor solution confirms only the presence of Pt NPs particles, which proves the successful synthesis of the Pt NPs. EDX also show presence of small wt % amounts of the performer halides that could be on the surface of PT NPs.

To investigate the morphology of nanomaterials prepared using a sunlight‐driven method, the pure Pt NPs synthesized from double vacancy perovskites, which appeared as spherical particles, were analyzed using a scanning electron microscope (SEM) (Figure S6). Moreover, EDX analysis was carried out for elemental analysis mapping (Figures S7 and S8), further revealing the homogeneous distribution of platinum within these particles. This information is fundamental and confirms these materials' composition and structural morphology.

The hydrodynamic size, polydispersity index (PDI), and zeta potential of the synthesized Pt NPs were determined using the dynamic light scattering (DLS) technique (Figures S9 and S10). Both zeta potential (a measure of surface charge) and PDI were considered to assess the colloidal stability of the as‐prepared NPs. Due to their high specific surface area and narrow size, Pt NPs are inherently prone to group in bigger clusters of NPs in water solution. Zeta potential measurements provide insight into their surface charge, which is critical for predicting the physical stability of NP suspensions. Large negative or positive zeta potential values generally indicate strong electrostatic repulsion between particles, minimizing the likelihood of flocculation. Conversely, low absolute values suggest insufficient repulsive forces to prevent aggregation, leading to a higher propensity for clustering. Generally, suspensions with zeta potential values between ±30 and ±40 mV are considered electrostatically stable of NPs [36].

For the sunlight‐driven synthesis investigated in this work, Pt NPs derived from Cs_2_PtCl_6_ exhibited a TEM size of 2.4 nm, a hydrodynamic diameter of 117.4 nm, a PDI of 0.260, and a zeta potential of ‐30.7 mV. Pt NPs obtained from Cs_2_PtBr_6_ showed a TEM size of 2.6 nm, a hydrodynamic diameter of 118.0 nm, a PDI of 0.288, and a zeta potential of −29.3 mV. These values indicate that the Cs_2_PtCl_6_‐derived Pt NPs are at the threshold of the commonly accepted stability criterion, whereas the Cs_2_PtBr_6_‐derived Pt NPs, with a slightly less negative zeta potential, may display comparatively lower electrostatic stabilization. Nevertheless, in both cases, the measured PDI values (< 0.3) suggest a moderately narrow size distribution, which is beneficial for maintaining consistent catalytic performance.

**FIGURE 3:**
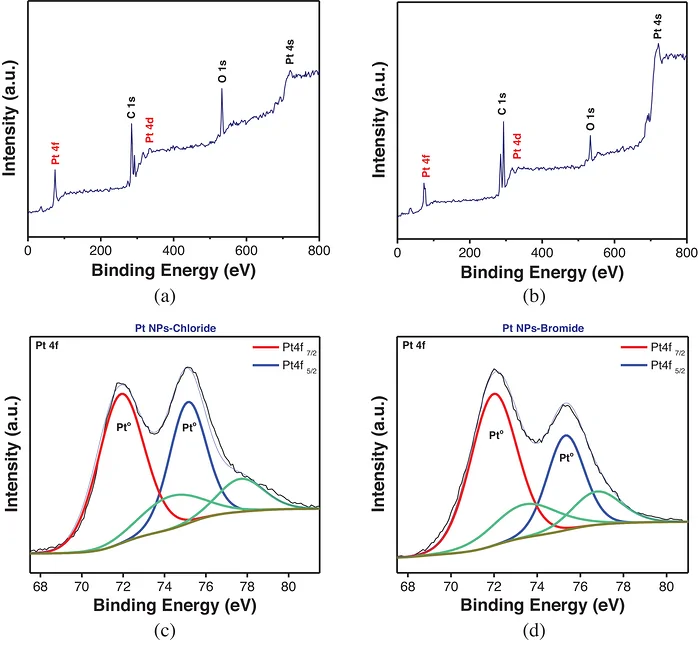
XPS analysis of platinum nanoparticles. (a) Survey spectrum and (c) Pt 4f regions for Pt NPs‐Chloride. (b) Survey spectrum and (d) Pt 4f regions for Pt NPs‐Bromide. The spectra show the characteristic peak fitting analysis is presented for the Pt 4f region, distinguishing between Pt^0^ (teal line) and Pt^2+^ states, providing insights into the chemical environment of Pt nanoparticles prepared from chloride and bromide forms.

The average TEM (Figure 2) and hydrodynamic sizes of the Pt NPs prepared using the sunlight‐driven method are 2.4–2.6 and 117–118 nm, respectively (Table S4). These sizes are attributed to the union of chlorine and bromine from the precursor methanolic extract, a product of the degradation of the initial perovskite, to the surface of the synthesized Pt NPs, which increases the value of the zeta potential.

x‐Ray photoelectron spectroscopy (XPS) confirms that both chloride‐ and bromide‐derived products expose predominantly metallic Pt surfaces (Figure 3). The survey spectra show intense Pt features (including Pt 4f/4d) together with C 1s and a weak O 1s signal (Figure 3a,b), while the C 1s maximum at 284.8 eV was used for charge referencing [37]. In the high‐resolution Pt 4f region (Figure 3c,d), the dominant doublet at ∼71.5/74.4 eV is characteristic of Pt^0^ [27, 38]. In contrast, smaller, higher‐binding‐energy components are consistent with Pt^2+^, arising during the aqueous methanol‐driven photoreduction of haloplatinate precursors, and can leave a small fraction of oxidized/hydroxylated species, indicating a metallic NP core with only partial surface passivation [30, 39, 40].

Subtle differences in lineshape between the chloride and bromide samples are plausibly linked to halide‐dependent surface chemistry [27] during growth and post‐synthesis processing, which can modulate the apparent Pt^0^/Pt^2+^ balance [40].

### 2.1 Photocatalytic MRR

The photocatalytic MRR activity of the Pt NPs samples was assessed over a 4 h reaction under simulated sunlight irradiation. Identical experiments carried out in the dark produced lower than the half of gas yields, (Figure S11), thereby confirming that the formation of methanol reforming by‐products arises also from photoinduced redox processes occurring at the NP surface. The gas evolution observed under dark conditions can be attributed to sonochemical effects induced by the 15‐min sonication used to degasify the reaction solution. This step, which enhances measurement accuracy by removing dissolved gases, has been shown to activate Pt NPs, enabling them to act as sonochemical catalysts for methanol reforming. This phenomenon is consistent with previous reports demonstrating sonochemical catalysis during methanol reforming [41, 42], resulting in significant hydrogen and other hydrocarbon gas production even in the absence of light (See Table S5).

**FIGURE 4:**
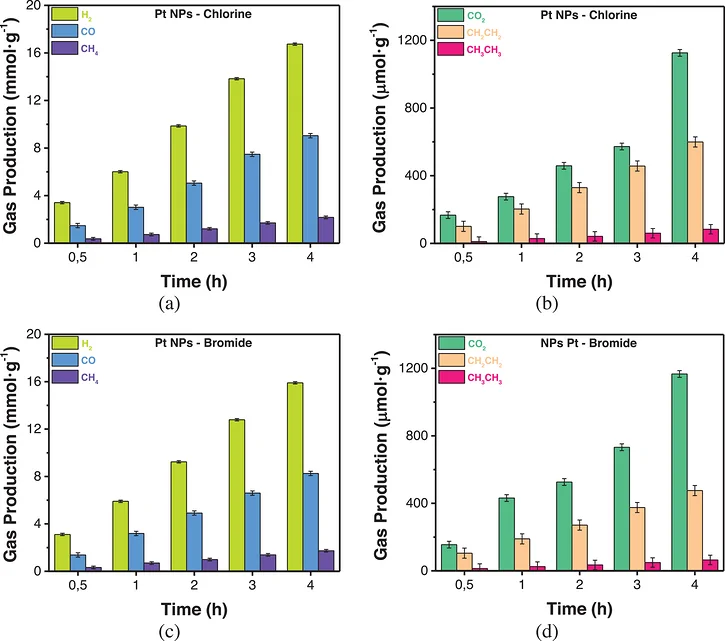
Histograms showing photocatalytic MRR over Pt (a, b) NPs‐chlorine and (c, d) NPs‐bromide under simulated sunlight irradiation: evolution of (a, c) H_2_, CO, CH_4_, and (b, d) CO_2_, CH_2_CH_2_, CH_3_CH_3_ products. Reaction conditions: Pt NPs (2 mg), *P*_Ar_ = 1.0 bar, at 4 h reaction time, simulated sunlight irradiation (130 mW cm^−2^), and temperature as indicated.

For Pt NPs derived from Cs_2_PtCl_6_ (Pt‐Chlorine), the temporal evolution of product formation (Figure 4a,b) revealed a steady increase in H_2_, CO, and CH_4_ production, reaching ∼16.8^−^, ∼8.6^−^, and ∼1.5 mmol·g^−1^, respectively, after 4 h. Concurrently, CO_2_, C_2_H_4_, and C_2_H_6_ formation reached approximately 1.18^−^, 0.95^−^, and 0.12 mmol·g^−1^, respectively. This product distribution indicates that the catalyst is highly effective at promoting both C─H bond activation and C─C coupling reactions, consistent with the multifaceted pathways of methanol photo reforming. Similarly, Pt NPs derived from Cs_2_PtBr_6_ (Pt‐Bromide) exhibited a comparable trend in gas evolution (Figure 4c,d), producing ∼16.0 mmol·g^−1^ H_2_, ∼8.0 mmol·g^−1^ CO, and ∼1.4 mmol·g^−1^ CH_4_ after 4 h, alongside CO_2_, C_2_H_4_, and C_2_H_6_ levels of ∼1.16^−^, 0.85^−^, and 0.10 mmol·g^−1^, respectively. Although the overall activity is slightly lower than that of the Pt‐Chlorine analogue, the difference is modest, suggesting that both perovskite‐derived catalysts exhibit excellent photocatalytic efficiency under identical reaction conditions (25°C, 1.0 bar Ar, 130 mW·cm^−2^).

Therefore, to determine the carbon balance, gas selectivity, and methanol conversion, we have included the following Equations (1)–(3) (see Supporting Information).

For the representative Pt NPs catalyst, a rigorous carbon mass balance yielded a recovery of 80%–83%. This remaining carbon is attributed to three primary factors. Firstly, minor fractions of low‐molecular‐weight hydrocarbons may partially redissolve in the aqueous medium, preventing their effective quantification via MicroGC. Secondly, gaseous methanol was clearly identified in the reaction headspace, as evidenced in the provided GC chromatograms (Figure S12), yet instrumentation limitations prevented its precise numerical quantification. Finally, liquid‐phase HPLC analysis detected only residual methanol, suggesting that any potential aqueous intermediates, such as formaldehyde or formic acid, are either formed or remain below the analytical limit of detection. These results demonstrate that the observed carbon deficit stems from unquantified gaseous species and trace‐level intermediates rather than fundamental mass loss, providing a comprehensive account of the carbon partitioning within the system.

Shown in Table S6 is the carbon‐based selectivity for all identified gaseous products under simulated sunlight irradiation. The results reveal a high selectivity toward CO (∼66%), suggesting that the direct deconstruction of methanol on the platinum surface is the primary pathway under sunlight irradiation. The inclusion of these values, alongside the carbon mass balance, ensures a more rigorous discussion of the catalytic performance.

This point is particularly relevant, as we have determined that selectivity is similar between light and dark conditions, with a slight difference. Under dark conditions, when the reaction is performed with hourly 15‐min sonication, methane selectivity increases by approximately 3%–5%, while CO selectivity decreases slightly. The selectivities for other gases remain essentially unchanged. These findings are corroborated in Table S7/, which shows the product selectivity under dark conditions. It is important to note that while methane selectivity increases slightly under dark conditions, the overall trends in product distribution do not change significantly, highlighting the influence of the sonication step in both conditions.

Post‑catalysis TEM analysis indicates that both Pt NPs‑chloride (Figure S13a,b) and Pt NPs‑bromide (Figure S13c,d) undergo a modest increase in average particle size following 4 h of methanol reforming under simulated sunlight. This increase is consistent with the general behavior of metal NPs under catalytic conditions, where particles tend to grow over time due to sintering or coalescence effects. This phenomenon has been previously reported: metal NPs tend to undergo size growth during prolonged catalytic activity, often due to thermal or reactive conditions [43, 44]. Notably, the XRD pattern (Figure S13e) of the recovered catalysts remains unchanged relative to the initial Pt NPs, confirming that the crystalline nature of the Pt NPs is preserved and no new bulk phases form during catalysis. These combined observations support the conclusion that, although minor size evolution occurs, the active Pt metallic phase remains structurally intact after the reforming reaction. Figure S14 shows that the Pt NPs photocatalyst retains substantial activity over five consecutive 4 h methanol reforming cycles under simulated sunlight. H_2_ remains the main product throughout, while CO and CH_4_ display the same overall trend. Gas yields decrease only slightly from the second to the 5th cycle, indicating good catalyst stability and minor deactivation under the applied conditions. However, the continued evolution of H_2_, CO, and CH_4_ confirms that the Pt NPs remain catalytically active after repeated use. This decrease may reflect the onset of gradual deactivation, associated with surface modification or partial loss of accessible active sites, and may also be partly related to progressive methanol consumption during recycling.

The higher H_2_ and CO yields observed for Pt‐Chlorine may be associated with its marginally higher absolute zeta potential (−30.7 mV) and slightly smaller TEM size (2.4 nm), which together enhance surface charge‐mediated stabilization and active site accessibility. In both cases, the monotonic rise in product concentrations over the reaction period indicates sustained catalytic activity without significant deactivation, underscoring the structural robustness and surface stability imparted by the sunlight‐driven synthesis.

The photocatalytic activities reported for methanol photoreforming over Pt‐based systems are summarized in Table 1, together with the results obtained in this work (entries 7 and 8). Direct comparison of literature data should be approached with caution, since reported rates depend strongly on reactor configuration (batch vs. continuous flow), irradiation source (UV vs. simulated sunlight), irradiance, catalyst mass, and loading of Pt NP co‐catalysts, carrier gas, and critically temperature.

**TABLE 1:** Photocatalytic activities reported in the literature for methanol photoreforming using Pt NPs‐based photocatalysts.

Entry	Catalyst	Reaction conditions	Gaseous products (mmol·g^−1^·h^−1^)		Reference
1	Pt/TiO_2_	Photocatalyst (800 mg), continuous‐flow, Xe–Hg lamp 200 W, UV (365 nm), 100°C–140°C, 2 h	**H_2_**: **CO_2_**: **CO**: **CH_4_**:	0.75 1.05 0.20 <1 × 10^−4^	[45]
2	Pt/TiO_2_	Photocatalyst (2.4 g), batch reactor, LED 10 W, UV (365 nm), 100°C, 4 h	**H_2_**: **CO_2_**: **CO**:	∼0.25 ∼0.06 ∼0.01	[46]
3	Pt‐containing TiO_2_ (one‐pot)	Photocatalyst (25 mg), batch reactor, LED (365 nm), 6.4 mW·cm^−2^, 100°C, 3 h	**H_2_**:	12	[47]
4	Flame‐made Cu‐Pt/TiO_2_	Photocatalyst (15 mg), batch reactor, 1.2 bar N_2_, Xe lamp 300 W (350–400 nm), 310 mW·cm^−2^ 40°C	**H_2_**:	22.7	[48]
5	Pt nanoclusters /g‐C_3_N_4_	Photocatalyst (10 mg), batch reactor, sunlight irradiation (Xe lamp 300 W), 25°C, 2 h	**H_2_**:	17.1	[49]
6	Flame‐spray‐pyrolysis Pt/TiO_2_	Photocatalyst (15 mg), continuous‐flow, Xe lamp 300 W, N_2_ atmosphere, 120 mW·cm^−2^, ∼3h	**H_2_**: **CO_2_**: **CO**:	19.5 2.06 0.06	[50]
7	Pt NPs‐chloride	Photocatalyst (2 mg) in MeOH/H_2_O (0.4 + 10 mL), batch reactor, 1.0 bar Ar, simulated sunlight irradiation (Xe–Hg lamp 150 W, 1.5 AM filter), 130 mW·cm^−2^, 25°C, 4 h	**H_2_**: **CO**: **CH_4_**: **CO_2_**: **C_2_^+:^**	4.2 2.3 0.5 0.3 0.17	This work
8	Pt NPs‐bromide		**H_2_**: **CO**: **CH_4_**: **CO_2_**: **C_2_^+:^**	4.0 2.1 0.4 0.3 0.1	This work

A clear trend nevertheless emerges: many of the highest H_2_ formation rates are achieved under conditions that depart substantially from ambient and/or rely on engineered supports. Conventional Pt/TiO_2_ benchmarks typically require UV irradiation and elevated temperatures (100°C–140°C), while enhanced performances are often linked to tailored composite architectures (e.g., Cu–Pt/TiO_2_ or Pt nanoclusters on g‐C_3_N_4_) or continuous‐flow operation (entries 1–6). These strategies can maximize photon utilization and charge separation, but at the cost of added materials complexity and, in several cases, harsher operating conditions.

In contrast, the Pt NP photocatalysts reported here (entries 7 and 8) operate under notably mild, application‐relevant conditions: batch operation at 25°C and 1 bar Ar under simulated sunlight (AM 1.5). Importantly, these activities are achieved without requiring a semiconducting support to enable catalysis, demonstrating that unsupported Pt NPs can directly drive methanol photoreforming under solar‐like irradiation. Beyond H_2_ evolution (∼4 mmol·g^−1^·h^−1^), measurable CO/CO_2_ formation and minor CH_4_ and C_2_^+^ products are observed, evidencing genuine reforming chemistry rather than simple hydrogen release. Taken together, these results highlight a simplified catalyst concept support free Pt NPs that can operate at room temperature under simulated sunlight, thereby lowering both material complexity and process severity relative to most Pt‐based systems reported to date.

### 2.2 Computational Studies

Using VASP (PAW‐PBE) with spin‐orbit coupling (SOC) and the Blöchl tetrahedron integration (full settings in the Supporting Information), we computed DOS/PDOS for Cs_2_PtX_6_ (*X *= Cl, Br) and its halide‐vacancy derivatives. Figure S15a,b shows a clean semiconductor with DOS(E_F_) = 0, consistent with the vacancy‐ordered double perovskite (A_2_BX_6_) literature and specific DFT reports on A_2_PtX_6_ (*X* = Cl, Br, I) [51, 52]. Introducing a halide vacancy (*V*_X_) creates an in‐gap state with strong Pt‐5d character (Figure S16a), in line with defect physics of halide perovskites/Vacancy‐Ordered Halide Perovskites where halide vacancies often act as deep traps (F‐center‐like) that localize carriers [53]. Upon adding one electron (photoinduced/donor case), that defect state becomes occupied (Figure S17a), *E*_F_ shifts upward, and finite DOS near *E*_F_ appears with Pt‐5d weight localized at the Pt adjacent to *V*_x_ an electronic signature of local Pt^+4^ reduction and concomitant weakening of Pt─Cl bonds in the incomplete [PtCl_5_]^−^ environment; coherent with the established photoreduction sequence of [PtCl_6_]^2−^ toward lower‐valent chloroplatinate species and ultimately Pt^0^ nuclei under light/methanol [54]. The bromide analogue shows the same qualitative trends but with a shallower vacancy level and a more readily populated DOS near *E*_F_ (Figures S16b–S17b), consistent with weaker Pt‐Br bonding and modified defect energetics that favor electron localization and reduction [55]. Collectively, Figures S10–S12 provide a compact computational rationale for sunlight‐driven Pt^0^ NP formation from A_2_PtX_6_ precursors: *V*_x_ creates a Pt‐centered in‐gap level; the photoelectron fills and localizes it, weakening Pt–X and triggering reduction, thereby supplying the minimal electronic pathway that precedes atom aggregation and NP nucleation, with a stronger propensity in Br than in Cl.

### 2.3 Proposed Mechanism of Production Pt NPs

The formation of Pt NPs from double‐vacancy halide perovskites, Cs_2_PtX_6_ (*X* = Cl^−^, Br^−^), proceeds via a light‐driven redox process rationalized as a coupled photoredox sequence guided by the semi‐reactions in Equations (1)–(6) (see for semi‐reactions). Initially, photoexcitation of Cs_2_PtX_6_ generates electron–hole pairs (hυ→h^+ ^VB + e^−^ CB), inducing charge separation and supplying reducing equivalents in the conduction band (Step 1). The photogenerated holes drive methanol oxidation, yielding reactive intermediates (e.g., CO, CO_2_) [41, 56] via hydrogen‐abstraction, producing methanol‐derived radicals and protons and protons (Step 2 and 3). In our system, headspace GC directly confirms carbon‐containing oxidation products, with CO formed at 2.26 mmol·g^−1^·h^−1^ and CO_2_ at 0.28 mmol·g^−1^·h^−1^, indicating that partial oxidation dominates over total oxidation under the present conditions; this selectivity is consistent with reported methanol photooxidation and reforming pathways. Concurrently, Pt (IV) species in PtX_6_2^−^ are reduced to Pt^0^ by methanol‐derived radicals [39] through a radical‐mediated, stepwise reduction pathway (Pt^4+^→Pt^2+^→Pt^0^), as supported by photochemical studies of PtX_6_^2^^−^ in methanol and consistent with the minor high‐binding‐energy Pt 4f component observed here [39, 57] (Steps 4 and 5), releasing HX as a by‐product due to progressive weakening of Pt─X bonds and concomitant X^−^/HX liberation. Finally, Pt^0^ atoms nucleate and grow into colloidal NPs (Step 6), facilitated by ongoing MRRs via an autocatalytic, surface‐mediated reduction process; the presence of water can accelerate early Pt─Pt bond formation and growth kinetics [58]. Moreover, halide‐dependent coordination and adsorption effects can modulate reduction kinetics and surface passivation, rationalizing the subtle Cl/Br‐dependent Pt 4f lineshape differences observed; related halide effects under irradiation have been reported for Cs_2_Pt(Cl, Br)_6_ [27]. This mechanism highlights the dual role of methanol as both a sacrificial reductant and a stabilizing agent during Pt NPs formation.

### 2.4 Semi‐Reactions

#### 2.4.1 Proposed Mechanism of Production Pt NPs

- Light absorption and charge separation
(1) $$Cs_{2}\mathrm{Pt}X_{6}+h\upsilon \to Cs_{2}\mathrm{Pt}{X_{6}}^{*}\left(h^{+}\mathrm{VB}+e^{-}\;\mathrm{CB}\right)$$
- Methanol oxidation by photogenerated holes
(2) $$CH_{3}\mathrm{OH}\to \mathrm{CO}+4H^{+}+4e^{-}\;\left(\mathrm{partial}\;\mathrm{oxidation}\right)$$
(3) $$CH_{3}\mathrm{OH}\to CO_{2}+6H^{+}+6e^{-}\;\left(\mathrm{total}\;\mathrm{oxidation}\right)$$
- Reduction of Pt (IV) species

(4)

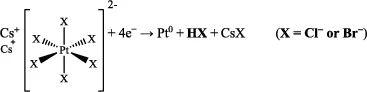

(5) $${\left[\mathrm{Pt}X_{6}\right]}^{2-}{+}^{•}CH_{3}\mathrm{OH}\to Pt^{0}+\mathrm{by}-\mathrm{products}$$
- Nucleation and growth of Pt NPs
(6) $$\eta Pt^{0}\to \mathrm{NPs}\;P{t^{0}}_{\eta }\left(\mathrm{growth}\right)$$

### 2.5 Photocatalytic Reaction Pathways

**FIGURE 5:**
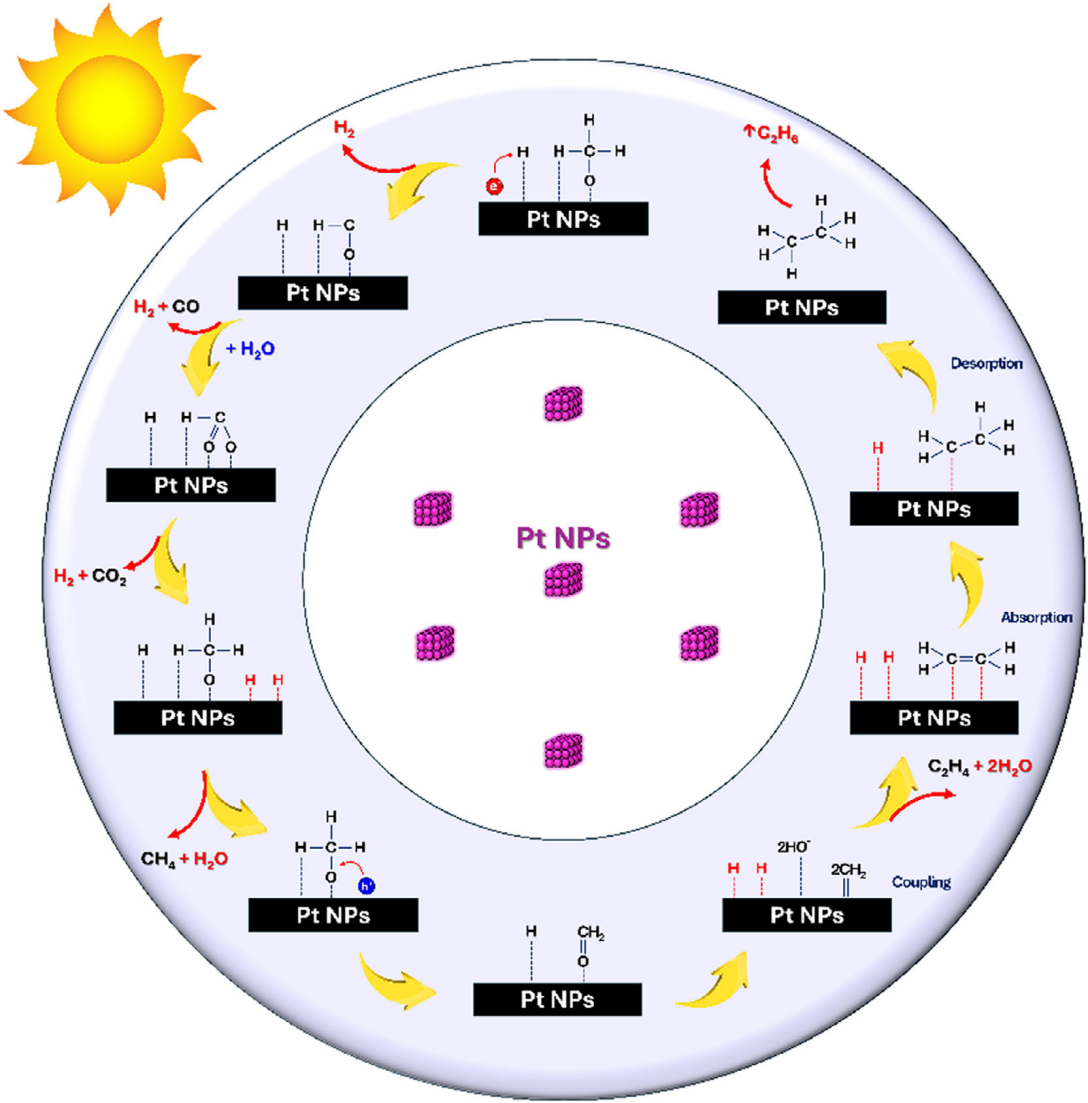
Mechanistic reaction pathways of methanol reforming over Pt NPs catalyst.

Under simulated sunlight, methanol can act as the electron/proton source while the metallic Pt surface provides sites for the methanol dehydrogenation, proton reduction, and subsequent CO hydrogenation/coupling chemistry (Figure 5). In this regard, based on the detected products, we have summarized in Table 2 the plausible series of reactions that occur during the catalytic experiments. First, methanol undergoes stepwise dehydrogenation on Pt to C_1_ surface intermediates and CO_(ad)_, which rationalizes the net “partial oxidation” half‐reaction CH_3_OH→CO+4H^+^+4e^−^ and the appearance of CO in the gas phase [59]. Further oxidation of CO_(ad)_ (and related C1 oxygenates) by water leads to CO_2_, consistent with CH_3_OH+H_2_O→CO_2_+6H^+^+6e^−^ [60]. The hydrogen‐evolution reaction consumes the released protons and electrons in these previous oxidative steps 2H^+^+2e^−^→H_2_, giving H_2_ as the dominant gaseous product under irradiation [61]. This sequence of reactions indicates the key role of water, providing an abundant proton reservoir for hydrogen evolution on Pt, being responsible for a fraction of the evolved H_2_ through light‐driven redox coupling with methanol oxidation [62]. Under anaerobic conditions, water can act as the terminal oxidant by supplying surface OH species that assist the further oxidation of C_1_ intermediates (including CO^∗^) to CO_2_, thereby helping to regenerate Pt sites [63]. These facts agree with the excess of H_2_ detected that overcomes the stoichiometric H_2_ produced due to methanol reforming.

**TABLE 2:** Proposed reaction steps and associated half‐reactions involved in methanol photoreforming over Pt NPs‐based catalysts, together with the main gaseous products formed under light irradiation.

Step	Type of reaction	Equation	Main Product
1	Partial oxidation	CH_3_OH → CO + 4H^+^ + 4e^−^	CO
2	Total oxidation	CH_3_OH + H_2_O → CO_2_ + 6H^+^ + 6e^−^	CO_2_
3	Reduction	2H^+^ + 2e^−^ → H_2_	H_2_
4	CO_2_ reduction	CO + 6H^+^ + 6e^−^ → CH_4_ + H_2_O	CH_4_
5	Coupling	2CH_2_ → C_2_H_4_	C_2_H_4_
6	C_2_H_4_ reduction	C_2_H_4_ + 2H^+^ + 2e^−^ → C_2_H_6_	C_2_H_6_

To confirm the proposed pathway, photocatalytic methanol reforming with isotope‐labelling experiments were performed to follow the origin of reaction products. In experiments with H_2_^18^O, fractions of CO/C_18_O and CO_2_/CO^18^O/C^18^O_2_ were detected, demonstrating the incorporation of water‐derived oxygen into the C1 oxidation products. (Figure S18) In separate D_2_O and CD_3_OD experiments, H_2_ remained detectable, while CD_3_OD produced CH_4_, C_2_H_4_, and C_2_H_6_ together with deuterated isotopes, evidencing ^1^H/^2^D transfer from methanol and water (Figure S19). Considering both cases, and reported studies, it is reasonable that this hydrogen and oxygen source is derived from OH* intermediates generated at Pt NPs [64].

Once the Pt NP surface gets rich in hydrogen, secondary hydrogenation pathways become more probable even under milder conditions. In particular, chemisorbed CO_(ad)_—formed directly from methanol decomposition—can be reduced stepwise to methane, matching CO+6H^+^+6e^−^→CH_4_+H_2_O, and providing a route to the observed CH_4_ [65]. In minor extend, C_2_ products can arise from CO reduction through the coupling of surface CH_x_ fragments generated during methanol decomposition (C–C bond formation) [66], yielding C_2_H_x_ intermediates that can desorb as ethylene (2CH_2_→C_2_H_4_). Finally, part of the ethylene is readily hydrogenated on Pt surface to ethane, consistent with C_2_H_4_+2H^+^+2e^−^→C_2_H_6_ [67].

## 3 Conclusion

In summary, this paper reports, for the first time, the green sunlight‐driven synthesis of Pt NPs from double‐vacancy halide perovskite precursors (Cs_2_PtCl_6_ and Cs_2_PtBr_6_) using simulated sunlight irradiation in aqueous‐methanol media. The structure, morphology, and colloidal stability of the Pt NPs synthesized via this sunlight‐driven approach were systematically characterized and compared. Both catalysts exhibited small particle sizes (2.4 nm for Cs_2_PtCl_6_‐derived Pt NPs and 2.6 nm for Cs_2_PtBr_6_‐derived Pt NPs) with narrow size distributions (PDI < 0.3) and zeta potential values indicative suitable colloidal stability.

Photocatalytic performance in the MRR demonstrated that both perovskite‐derived Pt NPs are highly efficient under simulated sunlight, producing significant quantities of H_2_, CO, and light hydrocarbons over a 4 h reaction period, while experiments conducted in the dark confirmed the exclusively photoinduced nature of the process. The monotonic increase in gas yields throughout the reaction suggests sustained catalytic activity without notable deactivation, highlighting the structural robustness imparted by the mild photochemical synthesis.

Overall, the sunlight‐driven method offers a sustainable and efficient route to Pt NPs with superior crystalline quality, controlled morphology, and strong photocatalytic performance. This methodology not only avoids the use of harsh reagents and high‐temperature treatments but also exploits the photochemical responsiveness of halide perovskites to yield high‐purity NPs. These findings establish sunlight‐assisted synthesis from double‐vacancy perovskites as a promising platform for the preparation of noble metal catalysts for solar‐to‐chemical energy conversion and other energy‐relevant applications.

## 4 Experimental Section

### 4.1 Chemicals

Perovskites (Cs_2_PtCl_6_ and Cs_2_PtBr_6_) were used as platinum precursors. Methanol (ACS reagent) was employed as a sacrificial electron donor, and Milli‐Q water was used for all experiments. All chemicals were used as received without further purification.

### 4.2 Instrumentation

The phase composition of the samples was determined by powder x‐ray diffraction (PXRD) on a Philips XPert diffractometer operating at 40 kV and 45 mA, with Ni‐filtered Cu Kα radiation. SEM images were acquired using a field emission FESEM (ZEISS Gemini SEM 500 instrument). The microstructure and morphology were observed through transmission electron microscopy (TEM) on a JEOL JEM‐2100F instrument operating at 200 kV. The hydrodynamic size, PDI, and the zeta potential of the NP dispersions were measured to quantify their colloidal stability, using a ZETASIZER Nano (Malvern Instruments). The chemical composition, elemental oxidation states and valence band of the samples were examined by XPS using a SPECS spectrometer with an MCD‐9 detector and a monochromatic Al Kα (1486.6 eV) x‐ray source; the C 1s peak at 284.8 eV served as the reference binding energy, and CASA XPS software was utilized for spectral deconvolution. The gaseous products were analysed by gas chromatography–mass spectrometry (GC–MS) in electron ionization (EI) mode using an Agilent 5977C MSD coupled to an Agilent 8890 GC system, equipped with an HP‐5MS UI capillary column (30 m × 0.25 mm × 0.25 µm).

### 4.3 Procedures

#### 4.3.1 Sunlight‐Driven Green Synthesis of Pt NPs

In a typical synthesis, 2.0 mg of Cs_2_PtCl_6_ (or Cs_2_PtBr_6_ for the bromide‐derived sample) was added to a 51 mL borosilicate glass reactor containing 10 mL of Milli‐Q water. Methanol (400 µL) was introduced as a sacrificial electron donor, and the suspension was magnetically stirred to ensure a homogeneous dispersion. The reaction vessel was purged with argon for 15 min to establish an inert atmosphere, then degassed for a further 15 min to release air molecules absorbed into water. It was subsequently purged with argon for another 15 min before irradiation. The suspension was then irradiated with a solar simulator at an incident power density of 130 mW·cm^−^^2^. This was conducted while being stirred continuously at 25°C for a period of 3 h. After the irradiation period, the reaction mixture developed a characteristic black color, indicative of Pt NP formation.

The crude black suspension was transferred to centrifuge tubes and centrifuged at 12,000 rpm for 10 min. The supernatant was discarded, and the resulting pellet was redispersed in Milli‐Q water. The wash/centrifugation cycle was repeated five times to remove residual methanol and soluble by‐products. After the final wash, the collected solid was oven‐dried at 50°C overnight to yield the Pt NPs as a dry powder. Washed colloidal samples intended for characterization were retained as aqueous suspensions and stored under argon at 4°C until analysis.

#### 4.3.2 Study of Reaction Parameters in the Synthesis of Pt NPs

To study how the size of Pt NPs varies, a reaction time of 4 h has been set, and the concentration of methanol in the aqueous solution has been varied (0.4, 1.6, and 2.8 mL), see Figures S1–S4. Furthermore, in all cases an irradiation power of 130 mW·cm^2^ was set, and although the reaction temperature was around 25°C, we measured with the thermal camera that the actual temperature in the irradiated liquid was approximately 36°C (Figure S20). We confirm that as the methanol concentration increased, the size of the Pt NPs also increased (Table S2). Experiments with aqueous solutions without methanol, under irradiation, generate Pt NPs but without control over the size distribution, accompanied by traces of large residual perovskite crystals, see Figure S21 to confirm this. Control experiments were carried out in which a perovskite water suspension was stirred for 23 h. Finally, it was observed that Pt NPs are produced only when the perovskite is irradiated; studies reported by Mego et al. describe that the photostability of platinum in water from perovskites can be monitored using inductively coupled plasma (ICP) analysis. Initial observations revealed that, in the absence of light, perovskite samples maintained a stable Pt^4+^ concentration for 3 days [27]. Irradiation of perovskite somehow increases its solubility in water.

#### 4.3.3 Photocatalytic MRR From Water Splitting (WS)

Perovskite suspension (Cs_2_PtCl_6_ and Cs_2_PtBr_6_) was prepared in a quartz reactor (51 mL) containing 2 mg of Pt NPs photocatalyst, 10 mL of Mili‐Q water and 400 uL methanol. Then, the reactor was subjected to sonication at 450 W for 15 min to obtain a good dispersion and later purged with argon during 30 min to evacuate air from inside. The suspended perovskites under stirring was irradiated with a commercially available Hg–Xe lamp (150 W, Hamamatsu ref L8253; Hamamatsu spotlight source L9566‐04 and light guide A10014‐50‐0110) with a commercially available AM 1.5G type filter (Lasing ref 81094) to obtain simulated sunlight irradiation. The measured irradiance when using the full spectrum of Hg–Xe lamp was 330 mW·cm^−^^2^, and this value decreased in the presence of an AM 1.5G filter to 130 mW·cm^−^^2^. The irradiances were measured using a commercially available photodiode (optical power meter 843‐R; Newport Corporation). Gaseous products of interest from these experiments were analyzed by connecting through the head space directly to the reactor with an Agilent 490 Micro GC system, equipped with TCD and FID detectors (Molsieve 5 Å column using argon as the carrier gas) without manual handling. During the experiment, the temperature was monitored, and the pressure was controlled by the manometer adapted to the photoreactor.

#### 4.3.4 Computational Methods and Computational Workflow

DFT calculations were performed with VASP 6.4 [68] using the PAW method [69] and the PBE‐GGA [70] functional, with explicit SOC due to 5d Pt. Standard PAW datasets from VASP were employed (recommended: Cs_sv, Pt_pv, Cl), ENCUT ≥ 520 eV, and ISYM = 0 under SOC. Brillouin‐zone sampling used Γ‐centered Monkhorst‐Pack meshes [71]: 6 × 6 × 6 for the 36‐atom conventional cell (SOC static) and 3 × 3 × 3 for the 2 × 1 × 1 supercell. DOS/PDOS were obtained with ISMEAR = −5 (Blöchl tetrahedron) [72], SIGMA = 0.05 eV, NEDOS = 3000; energies were plotted as *E*−*E*_F_ of each calculation PDOS projections used LORBIT = 11.

This study started from the conventional cell of Cs_2_PtCl_6_ (36 atoms), relaxed without SOC to **|**F**|**_max_ ≤ 0.02 eV Å^−1^ (PAW‐PBE, ENCUT ≥ 520 eV, ISMEAR = 0, SIGMA = 0.05, Γ‐centered 6 × 6 × 6 k‐mesh), then performed a SOC single‐point to obtain the bulk DOS (Figure S14). A 2 × 1 × 1 supercell (72 atoms) was built and a halide vacancy (*V*_X_) created by removing an apical *X* from a central [PtCl_6_] octahedron (defect fraction ≈ 2.1%), followed by SOC relaxation at fixed cell (ISIF = 2) and a SOC static DOS (Figure S15). To emulate a photoinduced electron, the same *V*_X_ model was run with NELECT = NELECT(V_X_) + 1 and a SOC static DOS (Figure S16). In all SOC statics we enforced NBANDS ≥ NELECT, used LASPH = .TRUE. and ISYM = 0, and NEDOS = 3000. The Cs_2_PtBr_6_ workflow was identical.

## Conflicts of Interest

The authors declare no conflicts of interest.

## Supporting Information

## Supporting information




**Supporting File**: chem71091‐sup‐0001‐SuppMat.pdf

## Data Availability

The data that support the findings of this study are available from the corresponding author upon reasonable request.
