# Supplementary material for: Sunlight‐Driven Green Synthesis of Platinum Nanoparticles From Double‐Vacancy Halide Perovskite Precursors for Methanol Reforming
Source: Chemistry. 2026 May 11;32(32):e03192. doi: 10.1002/chem.202503192 (PMC13505670; doi:10.1002/chem.202503192)
Supplement: Supplementary file 1 [file CHEM-32-e03192-s001.pdf]

Electronic Supporting Information

Sunlight-Driven Green Synthesis of Platinum Nanoparticles from Double-Vacancy Halide Perovskite Precursors for Methanol Reforming

Kevin Mego[1], Saray Chacón-Ruiz[1], Pedro Ruiz-Campos[2], Herme G. Baldoví[3] and Pedro Atienzar[1]**

[1] Instituto de Tecnología Química (ITQ), Consejo Superior de Investigaciones Científicas-Universitat Politècnica de València, 46022, Valencia, Spain.

E-mail: fkmegdel@itq.upv.es, scharui@itq.upv.es, pedatcor@itq.upv.es

[2] Universidad Científica del Sur, Panamericana Sur Km 19, 150142 Lima, Perú.

[3] Departamento de Química, Universitat Politècnica de València, Camino de Vera s/n, 46022 Valencia, Spain. E-mail: hergarba@itq.upv.es

*Corresponding author: pedatcor@itq.upv.es, hergarba@itq.upv.es

SUMMARY:

Table S1. Comparison of the previous studies for synthesis Pt NPs: Synthesis method, precursors, reducing/stabilizing agents, reaction time, reaction temperature and the sizes of the obtained Pt NPs. 6

Figure S1. HR-TEM image showing the effect of methanol concentration on Pt nanoparticle formation (NPs Pt-chloride, 1.6 mL MeOH). (a) Low magnification showing overall Pt nanoparticle morphology, (b) higher magnification illustrating nanoparticle structure and distribution, (c) high-resolution image revealing the crystal structure of Pt NPs, (d) particle size distribution from (b), with a mean of 4.33 ± 0.49 nm.. 7

Figure S2. HR-TEM image showing the effect of methanol concentration on Pt nanoparticle formation (NPs Pt-chloride, 2.8 mL MeOH). (a) Low magnification showing overall Pt nanoparticle morphology, (b) higher magnification illustrating nanoparticle structure and distribution, (c) high-resolution image revealing the crystal structure of Pt NPs, (d) particle size distribution from (b), with a mean of 6.48 ± 0.59 nm. 8

Figure S3. HR-TEM image showing the effect of methanol concentration on Pt nanoparticle formation (NPs Pt-bromide, 1.6 mL MeOH). (a) Low magnification showing overall Pt nanoparticle morphology, (b) higher magnification illustrating nanoparticle structure and distribution, (c) high-resolution image revealing the crystal structure of Pt NPs, (d) particle size distribution from (b), with a mean of 5.40 ± 0.68 nm. 8

Figure S4. HR-TEM image showing the effect of methanol concentration on Pt nanoparticle formation (NPs Pt-bromide, 2.8 mL MeOH). (a) Low magnification showing overall Pt nanoparticle morphology, (b) higher magnification illustrating nanoparticle structure and distribution, (c) high-resolution image revealing the crystal structure of Pt NPs, (d) particle size distribution from (b), with a mean of 6.78 ± 0.70 nm. 9

Table S2: Overview of Pt Nanoparticle Size Distribution and Formation Conditions. This table highlights the effect of varying methanol concentrations (1.6 mL and 2.8 mL) on the formation and size distribution of Pt nanoparticles. 9

Figure S5. Energy-dispersive X-ray (EDX) of Pt NPs synthesized from perovskites: (a) Cs_2PtCl_6 and (b) Cs_2PtBr_6 precursor. 10

Table S3. EDX quantification of the element normalized. 10

Figure S6. Scanning electron microscope (SEM) images of Pt NPs synthesized from perovskites: (a) Cs_2PtCl_6 and (b) Cs_2PtBr_6 precursor.. 11

Figure S7. FESEM image and EDX elemental mapping analyses of Pt NPS synthesized from Cs_2PtCl_6 perovskite. 11

Figure S8. FESEM image and EDX elemental mapping analyses of Pt NPS synthesized from Cs_2PtBr_6 perovskite 11

Figure S9. Hydrodynamic light scattering (DLS) of Pt NPs synthesized from perovskites: (a) Cs_2PtCl_6 and (b) Cs_2PtBr_6 precursor. 12

Figure S10. The averaged (over three measurements) zeta potential distribution for colloidal platinum nanoparticles: (a) NPs Pt-Chloride and (b) NPs Pt-Bromide. Sample concentration was 0.2 mg/mL in 10 mL Milli-Q water with a pH value of 5. 12

Table S4. TEM average size and hydrodynamic light scattering (DLS) of Pt NPs synthesized by sunlight-driven method. 12

Figure S11. Gas evolution profiles for Pt NPs–chloride: (a) H_2, CO, CH_4 and (b) CO_2, C_2H_4, C_2H_6; and Pt NPs–bromide: (c) H_2, CO, CH_4 and (d) CO_2, C_2H_4, C_2H_6. Dark conditions: Pt NPs (2 mg), P_{Ar} = 1.0 bar, at 4h reaction time and room temperature. ... 13

Table S5. Gas-phase products evolved under dark conditions with and without degasification for Pt NPs-chlorine and Pt NPs-bromide catalysts. The table compares gas production (H_2 and hydrocarbons) with and without a 15-minute sonication step to degas the reaction solution, providing insights into sonochemical activity. 13

Figure S12. Representative gas chromatograms (GC) illustrating the reaction product profile obtained during methanol reforming. Both Pt (a) NPs-chlorine and (b) NPs-bromide present the MS5A column output (top) identifying hydrogen and carbon monoxide, and the PPQ column output (bottom) showing methane, carbon dioxide, light hydrocarbons, and gas phase methanol. 15

Table S6. Catalytic performance and product selectivity for the sunlight-driven methanol reforming reaction. 16

Table S7. Product selectivity for the dark conditions methanol reforming reaction. 16

Figure S13. HR-TEM images and particle size distribution of platinum nanoparticles after methanol reforming reaction for 4 hours under simulated sunlight irradiation. (a, b) Pt NPs-chloride with a mean size of 3.4 nm. (c, d) Pt NPs-bromide with a mean size of 3.8 nm. (e) XRD pattern of Pt NPs after methanol reforming at 4 hours of reaction, showing the crystallinity of the nanoparticles post-catalysis. 17

Figure S14. Recycling of the photocatalyst NPs-Pt: evolution of (a) H_2, (b) CO, (c) CH_4. Reaction conditions: photocatalyst (2mg), P_{Ar} = 1.0 bar, at 4h reaction time, simulated sunlight irradiation (130 mW cm^{-2}), and room temperature. 18

Figure S15. Total DOS of Cs_2PtX_6 (bulk) computed with PAW–PBE + SOC; energies plotted as $E-E_F$: (a) Cs_2PtCl_6 and (b) Cs_2PtBr_6. Both are semiconductors with DOS(E_F) = 0; Cs_2PtBr_6 shows a smaller E_g. 18

Figure S16. DOS with a single halide vacancy (V_X); energies plotted as $E- E_F$: (a) Cs_2PtCl_6 and (b) Cs_2PtBr_6. V_X introduces a narrow in-gap state near E_F with strong Pt-5d character at the Pt adjacent to V_X; in Br this donor lies closer to E_F. 18

Figure S17. DOS for $V_X + e^-$ (one excess electron per supercell); energies plotted as $E- E_F$: (a) Cs_2PtCl_6 and (b) Cs_2PtBr_6. The vacancy-centred level becomes occupied, yielding finite DOS at E_F and localized Pt-5d weight. 19

Figure S18. GC–MS spectra after photocatalytic methanol reforming over Pt NPs under isotope-labelling conditions, using $H_2{}^{18}O$ experiment. Panels show the signals assigned to (a) CO, and (b) CO_2, together with their corresponding $C^{18}O$, $CO^{18}O$ and $C^{18}O_2$ isotopes. 19

Figure S19. GC–MS spectra of the gaseous products formed after photocatalytic methanol reforming over Pt NPs under isotope-labelling conditions, using D_2O and CD_3OD in separate experiments. Panels show the signals assigned to (a) H_2, (b) CH_4, (c) C_2H_4, and (d) C_2H_6, together with their corresponding deuterated isotopes. 20

Figure S20. Surface temperatures of the quartz reactor were monitored using an infrared thermal camera (HIKMICRO SuperIR). The temperature readings ranged from 26.8 °C to 36.2 °C, with the highest measured at the centre of the reactor, providing insights into the thermal distribution during the experiment. 20

Figure S21. FESEM analysis of (a) Cs_2PtCl_6 and (b) Cs_2PtBr_6 under light irradiation during photocatalysis in water. The images show the surface morphology of the perovskites after prolonged light irradiation (t = 23 h). Scale bar: 200 nm. Retrieved from Mego et al., *Adv. Energy Sustain. Res.*, 2025.[12] 21

Supplemental Text

Electronic-structure rationale for light-driven Pt^0 nucleation from Cs_2PtX_6 (X= Cl, Br) (DOS/PDOS and defect-state occupation)

The calculated DOS set (PAW–PBE+SOC, energies plotted as $E - E_F$) captures a minimal, common pathway to Pt^0 in both halides. For Cs_2PtCl_6 the pristine bulk shows DOS(E_F)=0, with the valence band spanning ≈ -1.6 to -0.2 eV and the conduction onset at ≈ +2.0 – 2.2 eV (Fig. S8a), consistent with vacancy-ordered double perovskites (VODPs)[1]. Introducing a halide vacancy (Fig. S9a) yields a narrow in-gap level pinned near 0 eV (a peak at E_F) while the CBM remains high; this defect peak carries strong Pt-5d character at the Pt adjacent to V_X, in line with deep F-center-like traps in halide lattices[2] that capture photogenerated electrons. Upon adding one electron ($V_X + e^-$) (Fig. S10a), the defect level becomes occupied and remains pinned at E_F (DOS($E_F > 0$)), indicating local $Pt^{+4} \rightarrow Pt^{+3}$ reduction and weakening of Pt–Cl bonds in the $[PtCl_5]^-$ environment via occupation of antibonding d-states. Under continuous light and methanol, that center progressed to Pt^{+2} and Pt^0, enabling Pt-atom diffusion/coalescence and nanoparticle nucleation, in agreement with the established photoreduction sequence of $[PtCl_6]^{-2}$ in alcoholic media with the alcohol as sacrificial donor.[3,4]

The bromide analogue follows the same progression: pristine Cs_2PtBr_6 remains semiconducting (DOS(E_F)=0) with a smaller gap (E_g ≈ 1.0 – 1.2 eV, Fig. S8b). V_x generates an in-gap donor even closer to E_F (Fig. S9b), and upon adding one electron ($V_x + e^-$) a more readily populated DOS at E_F emerges (Fig. S10a), evidencing easier electron trapping and local Pt reduction accompanied by Pt–Br weakening in $[PtBr_5]^-$. Collectively, Figs. S8-S10 show that bulk → V_X → $V_X + e^-$ creates a Pt-centered in-gap state whose occupation (finite DOS at E_F) is the decisive electronic trigger for $Pt^{+4} \rightarrow Pt^0$ in both halides-with a stronger in Br due to its shallower vacancy level and lower CBM, which reduce the energetic cost for electron localization and thereby accelerate Pt^0 nucleation.

Table S1. Comparison of the previous studies for synthesis Pt NPs: Synthesis method, precursors, reducing/stabilizing agents, reaction time, reaction temperature and the sizes of the obtained Pt NPs.

**Synthesis method**	**Precursors**	**Reducing/stabilizing agents**	**Reaction time**	**Reaction temperature**	**Size (nm)**	**Ref.**
Chemical reduction	$PtCl_4$	$NaBH_4$ / PVP	-	Room Temp. (~25 °C)	2.5-8.5	5
Green synthesis	H_2PtCl_6	Quercus glauca leaves	30 min	80 °C	5-15	6
Microbial biosynthesis	H_2PtCl_6	Gram-negative and Gram-positive bacteria	1 h	90 °C	2.8	7
Sonication	K_2PtCl_4	Prosopis farcta fruits	30 min	Room Temp. (25 °C)	3.5	8
Hydrothermal	K_2PtCl_6	PVP	8 h	160 °C	2.5	9
Laser ablation	Pt (metallic)	-	40 min	Room Temp. (~25 °C)	2.2-2.8	10
Microemulsion	H_2PtCl_6	MeOH / $NaBH_4$ / PVP	-	-	12-14	11
Sunlight-driven	Cs_2PtCl_6	MeOH	3 h	Room Temp. (25 °C)	2.4	This work
Sunlight-driven	Cs_2PtBr_6	MeOH	3 h	Room Temp. (25 °C)	2.6	This work

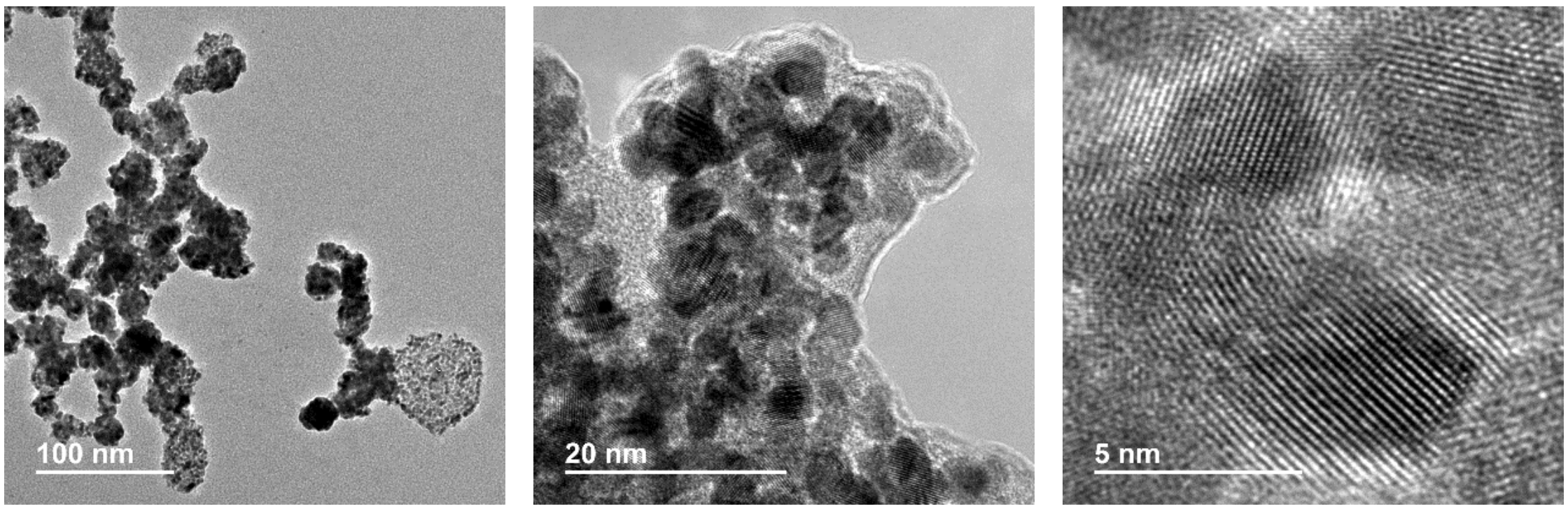

(a) (b) (c)

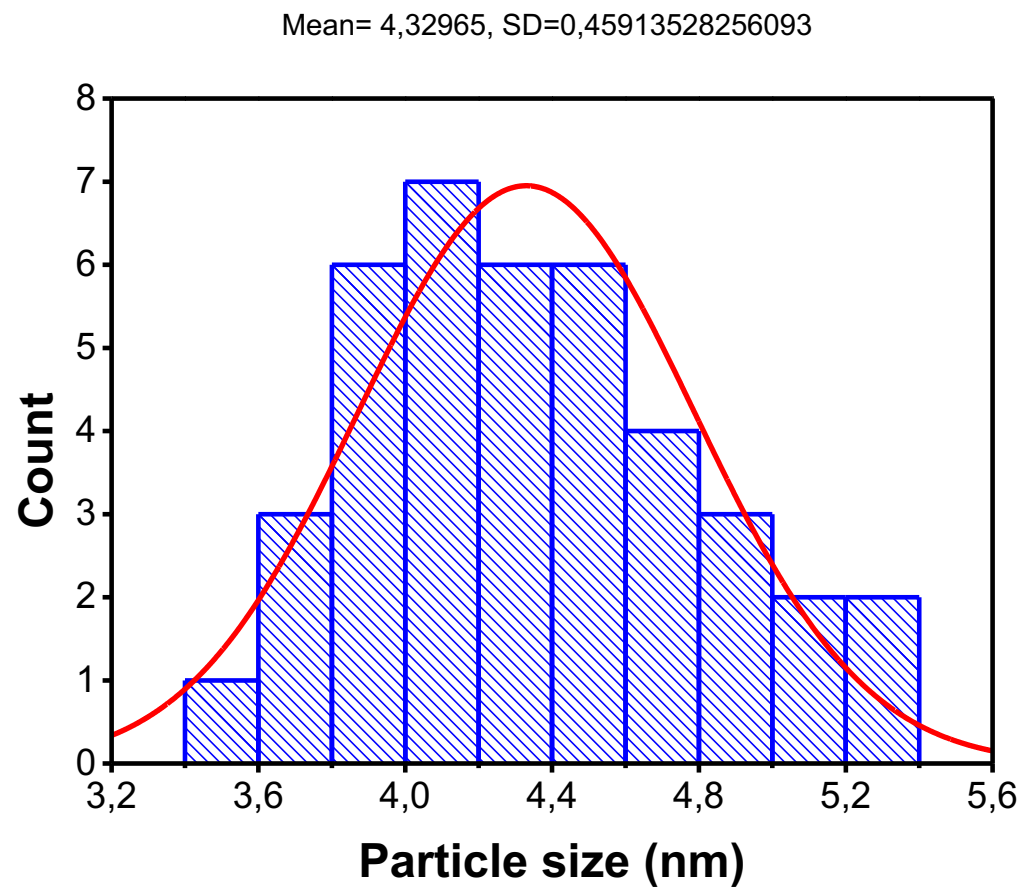

(d)

Figure S2. HR-TEM image showing the effect of methanol concentration on Pt nanoparticle formation (NPs Pt-chloride, 1.6 mL MeOH). (a) Low magnification showing overall Pt nanoparticle morphology, (b) higher magnification illustrating nanoparticle structure and distribution, (c) high-resolution image revealing the crystal structure of Pt NPs, (d) particle size distribution from (b), with a mean of 4.33 ± 0.49 nm.

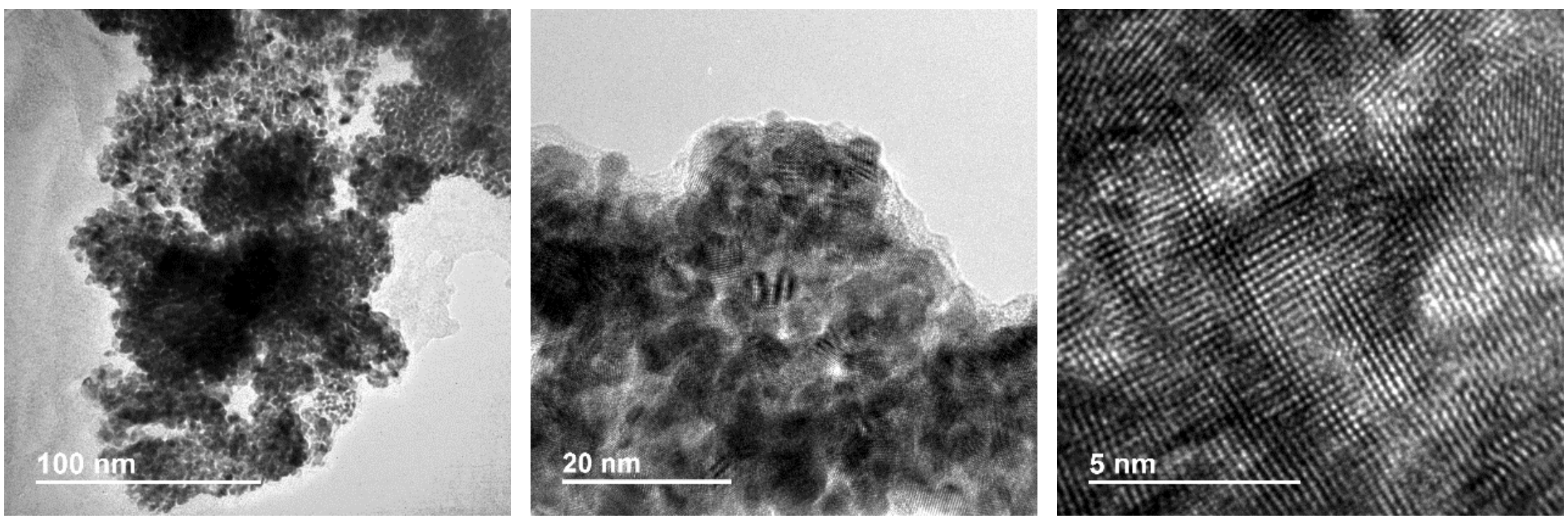

(a) (b) (c)

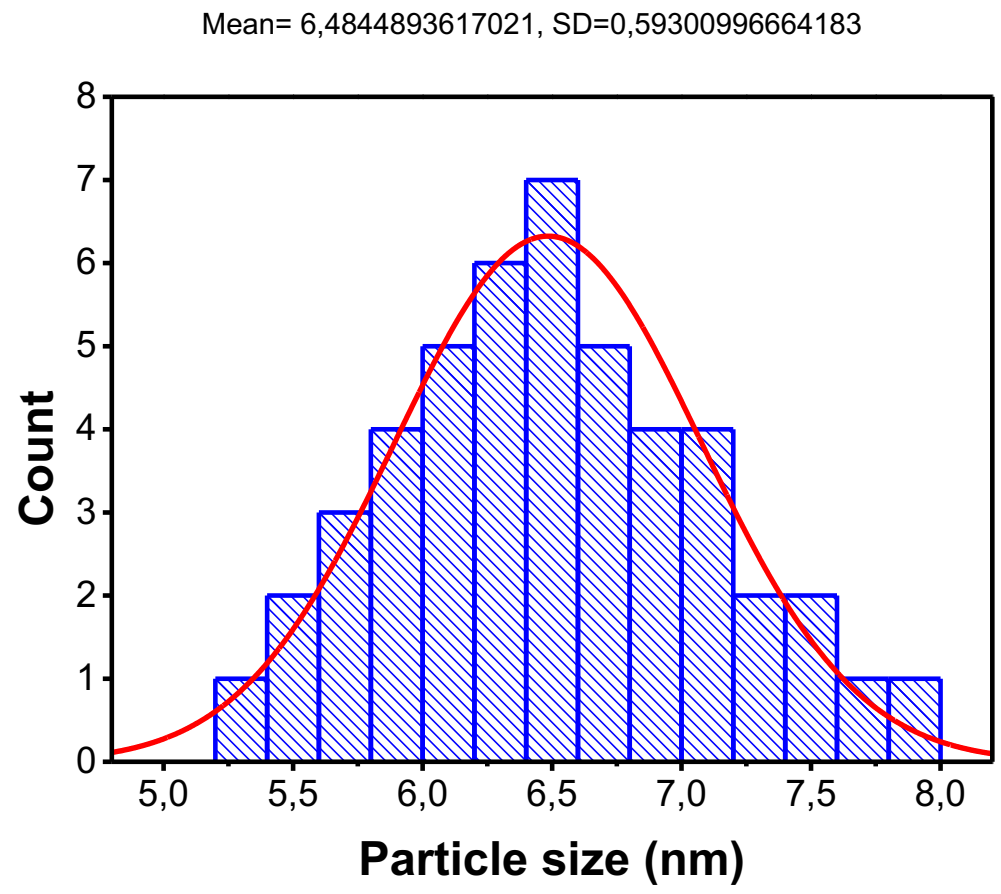

(d)

Figure S3. HR-TEM image showing the effect of methanol concentration on Pt nanoparticle formation (NPs Pt-chloride, 2.8 mL MeOH). (a) Low magnification showing overall Pt nanoparticle morphology, (b) higher magnification illustrating nanoparticle structure and distribution, (c) high-resolution image revealing the crystal structure of Pt NPs, (d) particle size distribution from (b), with a mean of 6.48 ± 0.59 nm.

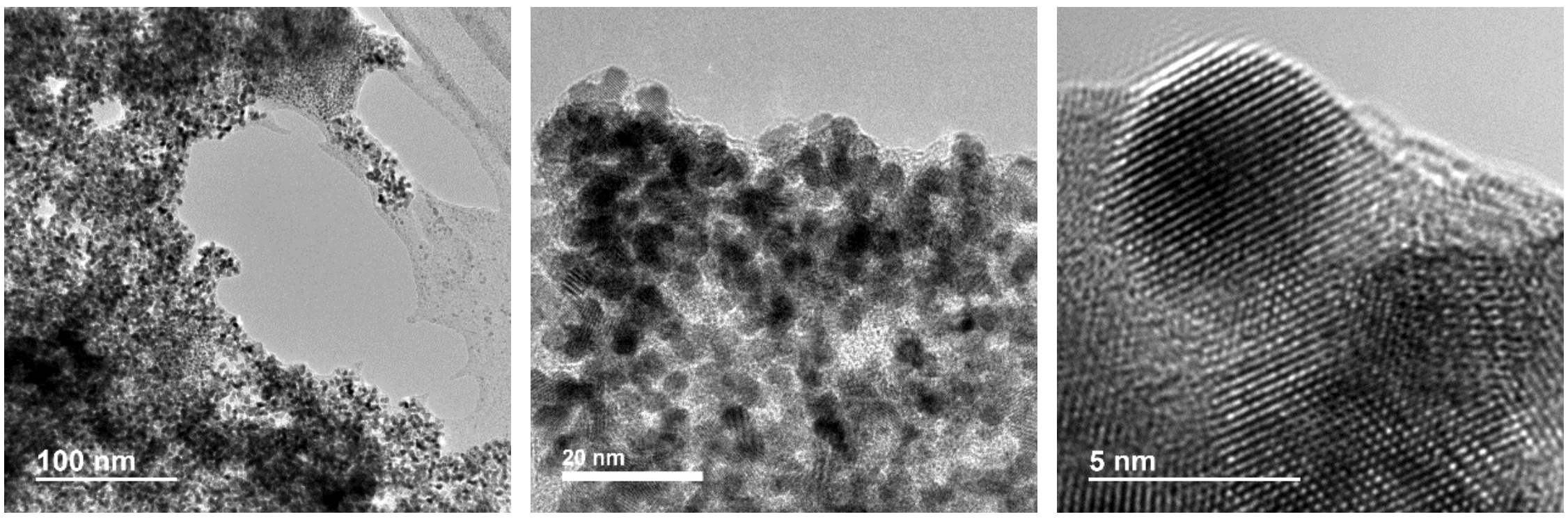

(a) (b) (c)

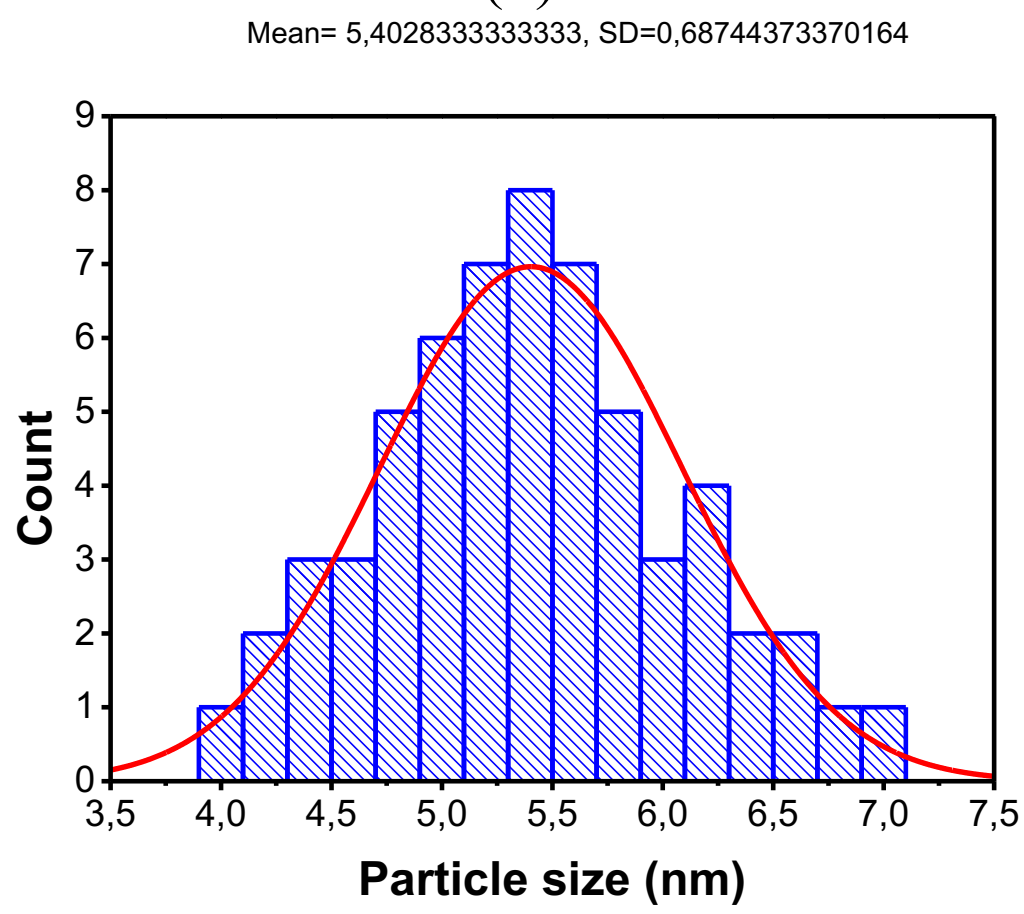

(d)

Figure S4. HR-TEM image showing the effect of methanol concentration on Pt nanoparticle formation (NPs Pt-bromide, 1.6 mL MeOH). (a) Low magnification showing

overall Pt nanoparticle morphology, (b) higher magnification illustrating nanoparticle structure and distribution, (c) high-resolution image revealing the crystal structure of Pt NPs, (d) particle size distribution from (b), with a mean of 5.40 ± 0.68 nm.

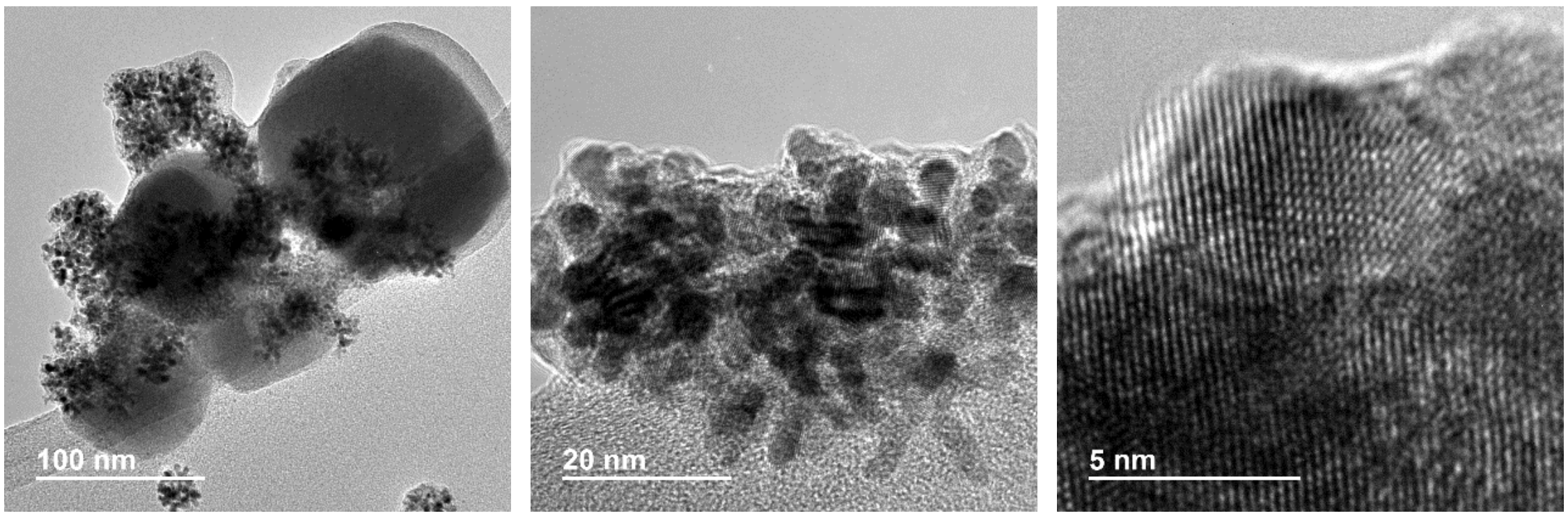

(a) (b) (c)

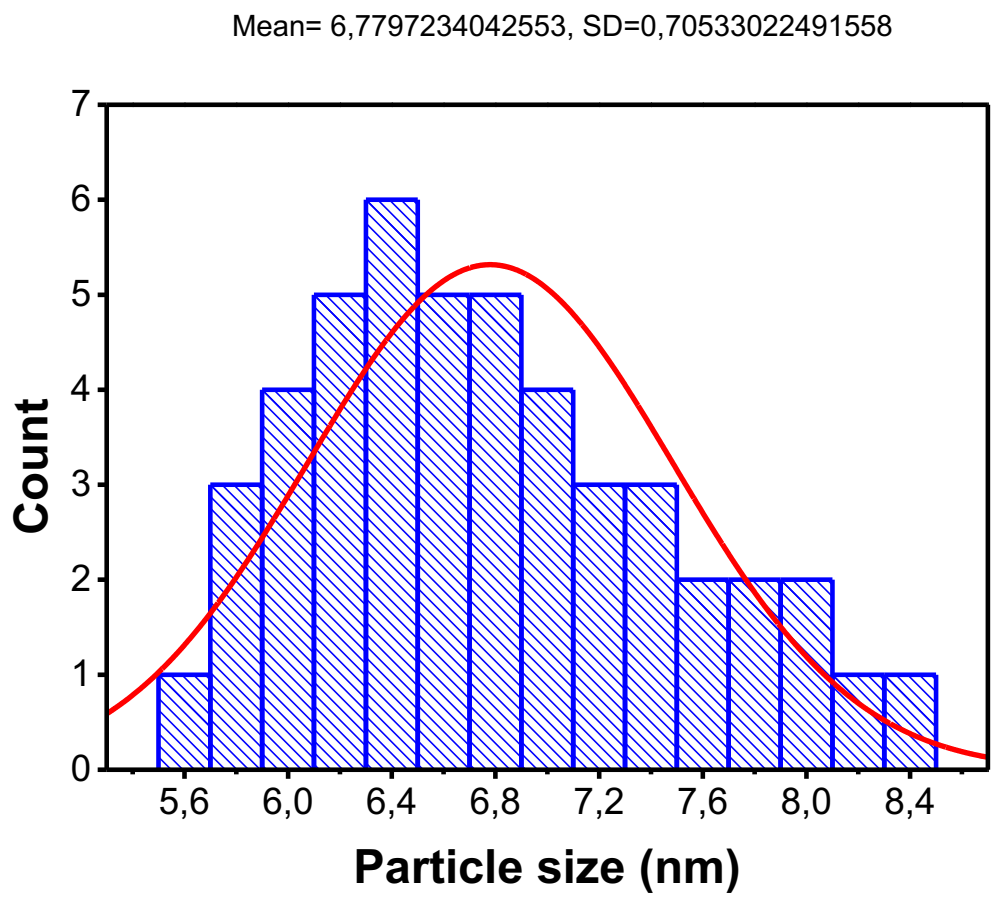

(d)

Figure S5. HR-TEM image showing the effect of methanol concentration on Pt nanoparticle formation (NPs Pt-bromide, 2.8 mL MeOH). (a) Low magnification showing overall Pt nanoparticle morphology, (b) higher magnification illustrating nanoparticle structure and distribution, (c) high-resolution image revealing the crystal structure of Pt NPs, (d) particle size distribution from (b), with a mean of 6.78 ± 0.70 nm.

Table S6: Overview of Pt Nanoparticle Size Distribution and Formation Conditions. This table highlights the effect of varying methanol concentrations (1.6 mL and 2.8 mL) on the formation and size distribution of Pt nanoparticles.

Catalyst	Size	[MeOH] mL
	2.4 ± 0.45	0.4
Pt- chloride	4.3 ± 0.46	1.6
	6.5 ± 0.59	2.8
Pt- bromide	2.6 ± 0.35	0.4
	5.4 ± 0.68	1.6
	6.8 ± 0.70	2.8

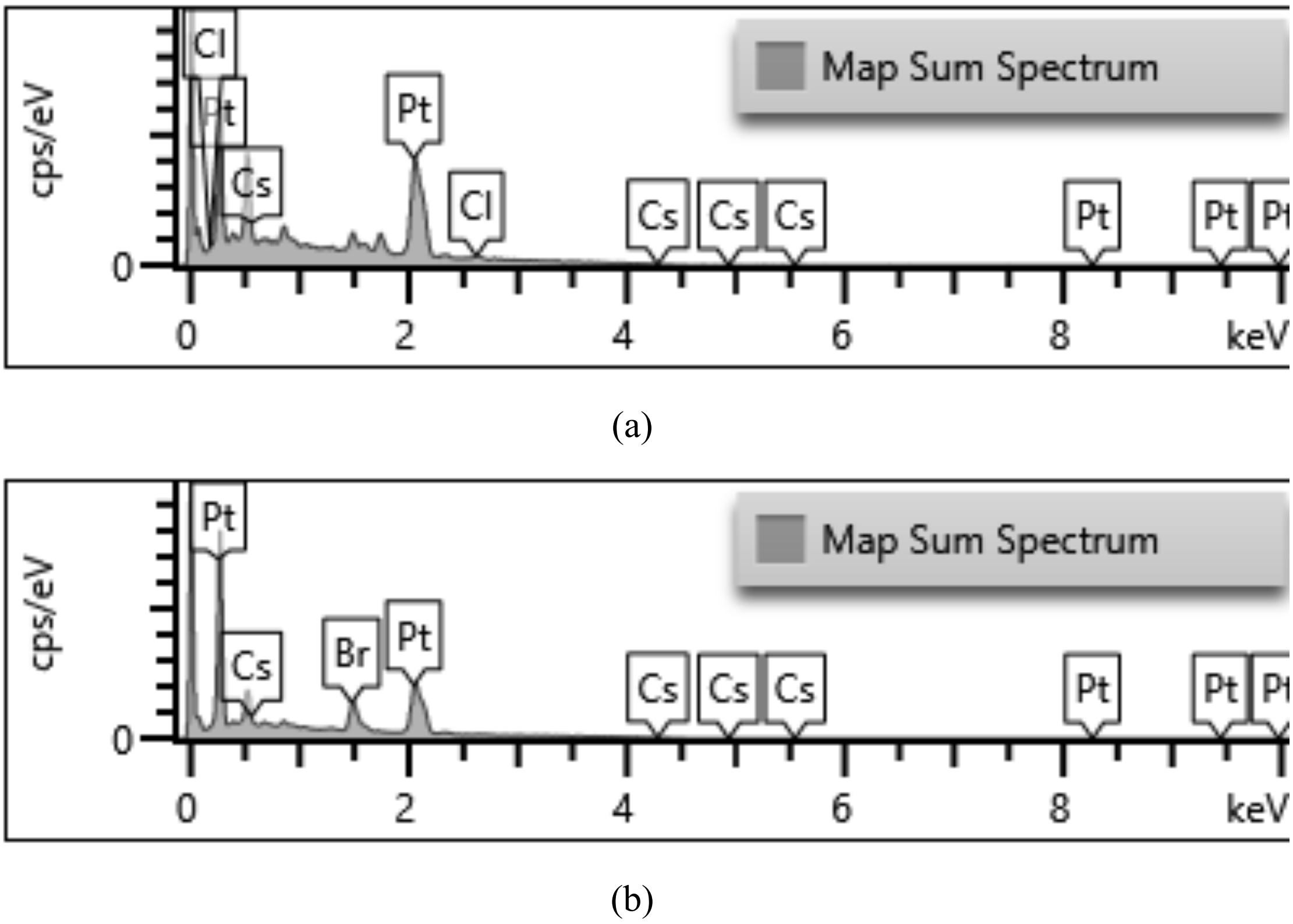

Figure S7. Energy-dispersive X-ray (EDX) of Pt NPs synthesized from perovskites: (a) Cs_2PtCl_6 and (b) Cs_2PtBr_6 precursor.

Table S8. EDX quantification of the element normalized.

**Pt NPs-Chloride method**			**Pt NPs-Bromide method**		
**Elements**	**Line Type**	**Weight (%)**	**Elements**	**Line Type**	**Weight (%)**
Pt	M	97.72	Pt	M	89.68
Cl	K	2.28	Br	L	10.32
Total		100	Total		100

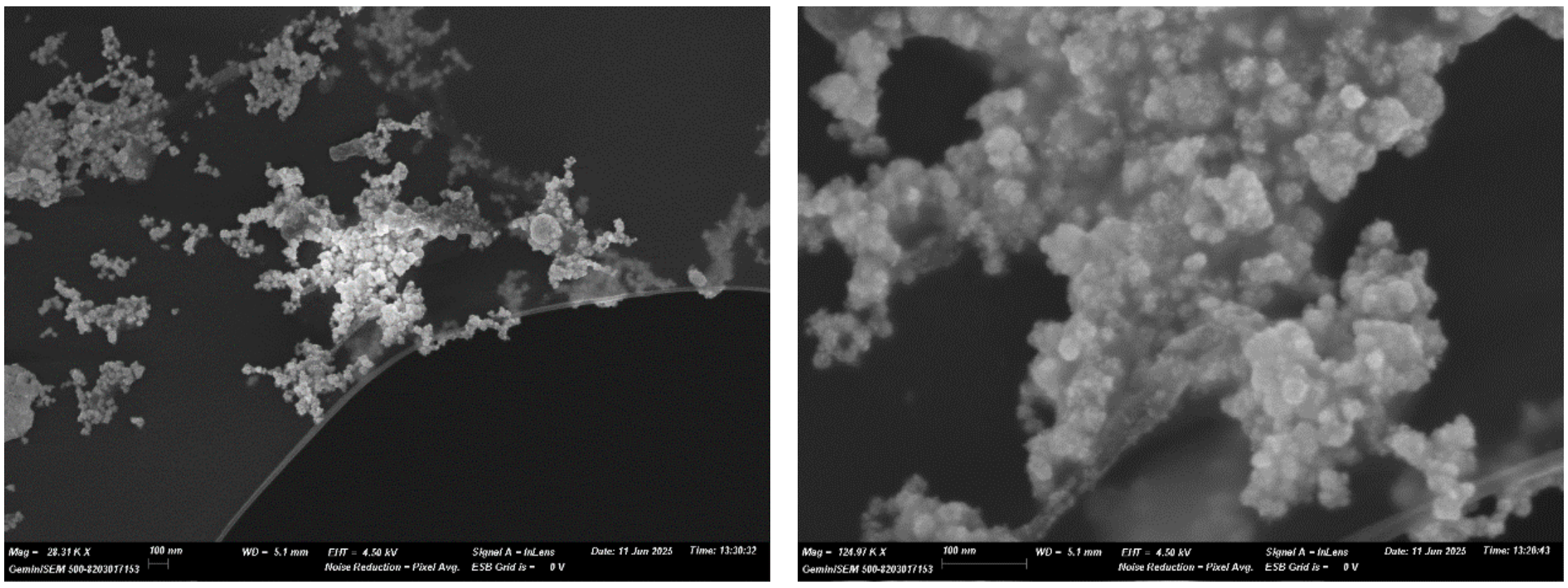

(a) (b)

Figure S9. Scanning electron microscope (SEM) images of Pt NPs synthesized from perovskites: (a) Cs_2PtCl_6 and (b) Cs_2PtBr_6 precursor.

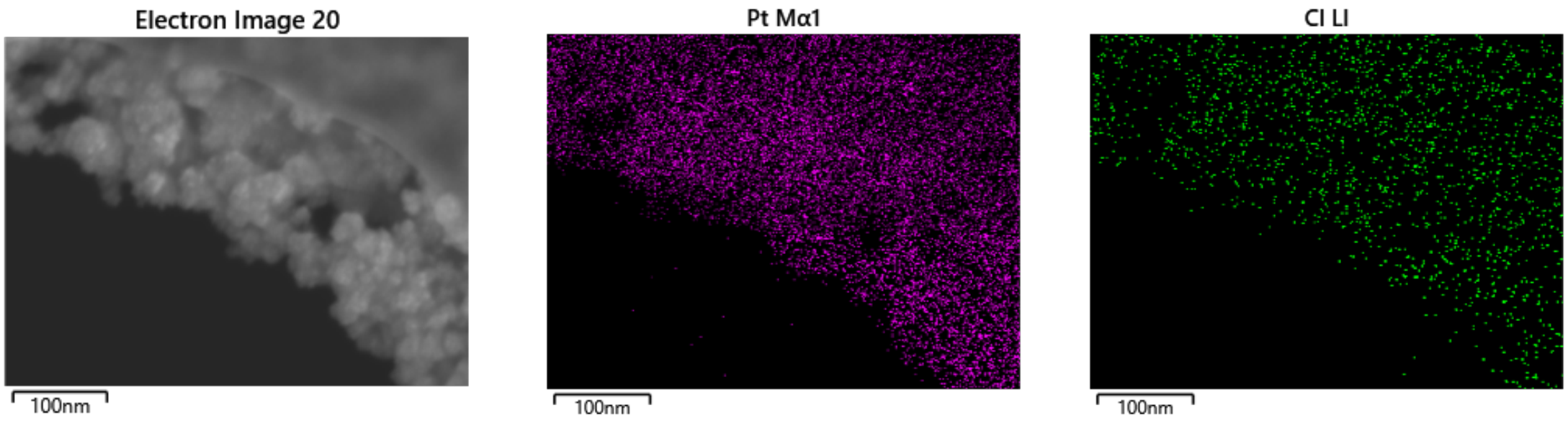

Figure S10. FESEM image and EDX elemental mapping analyses of Pt NPS synthesized from Cs_2PtCl_6 perovskite.

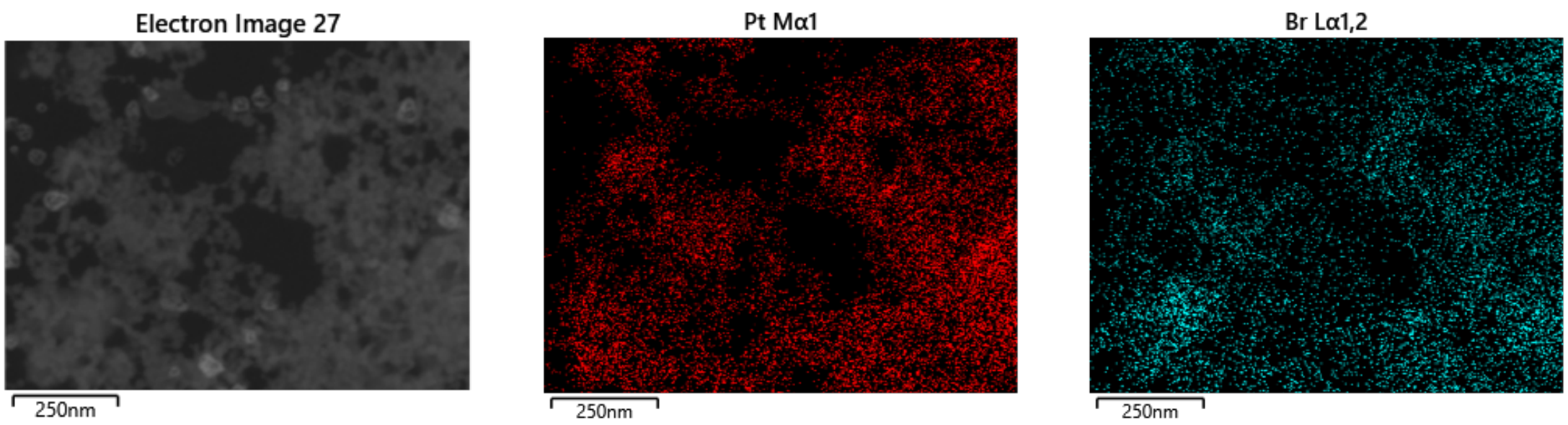

Figure S11. FESEM image and EDX elemental mapping analyses of Pt NPS synthesized from Cs_2PtBr_6 perovskite.

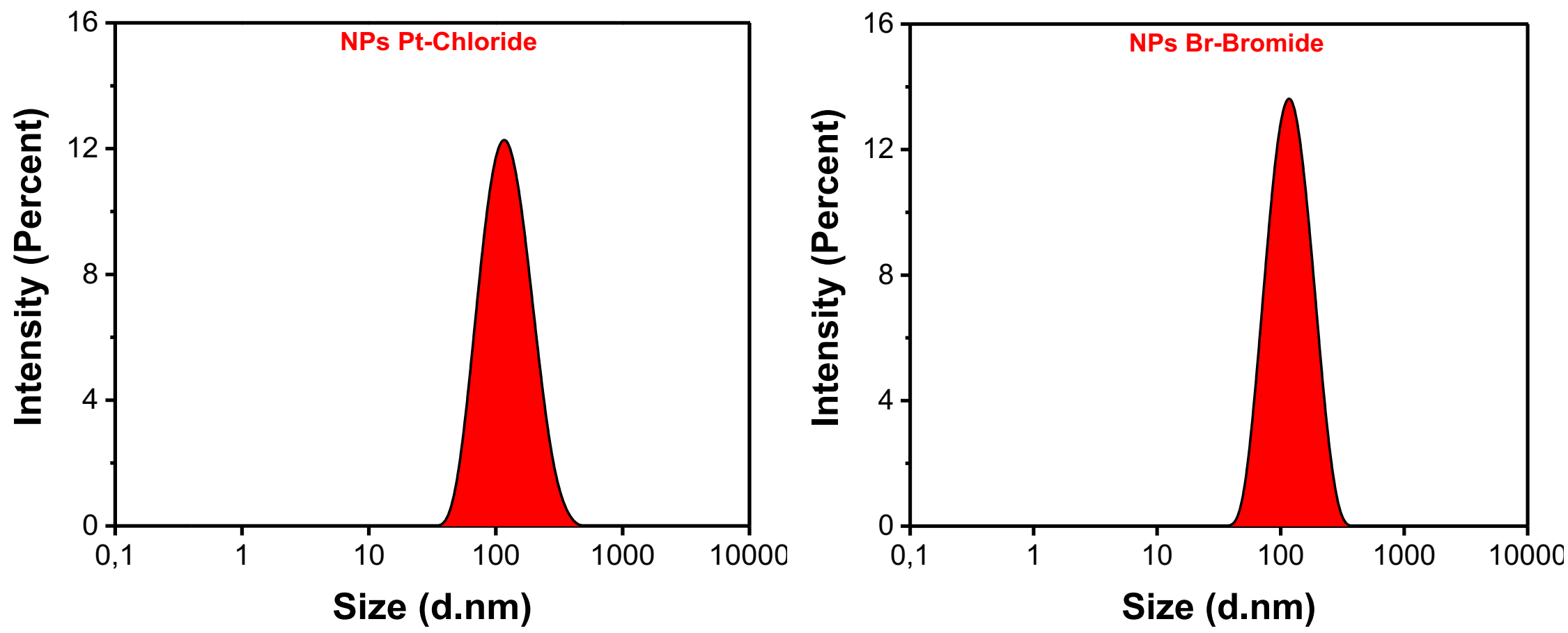

(a) (b)

Figure S12. Hydrodynamic light scattering (DLS) of Pt NPs synthesized from perovskites: (a) Cs_2PtCl_6 and (b) Cs_2PtBr_6 precursor.

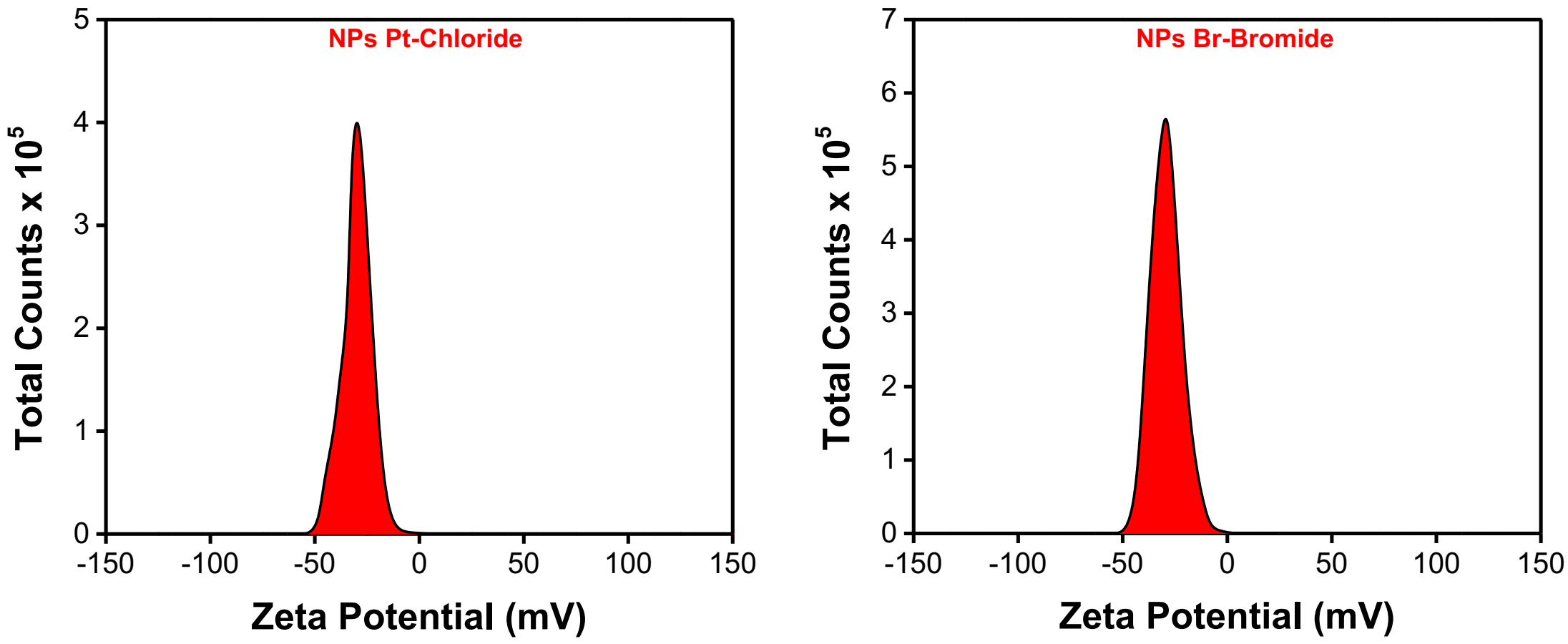

(a) (b)

Figure S13. The averaged (over three measurements) zeta potential distribution for colloidal platinum nanoparticles: (a) NPs Pt-Chloride and (b) NPs Pt-Bromide. Sample concentration was 0.2 mg/mL in 10 mL Milli-Q water with a pH value of 5.

Table S14. TEM average size and hydrodynamic light scattering (DLS) of Pt NPs synthesized by sunlight-driven method.

Method	Precursor	TEM average size (nm)	Hydrodynamic size (nm)	PDI	Zeta potential (mV)
Sunlight-	Cs_2PtCl_6	2.4	117.4	0.260	-30.7
driven	Cs_2PtBr_6	2.6	118.0	0.288	-29.3

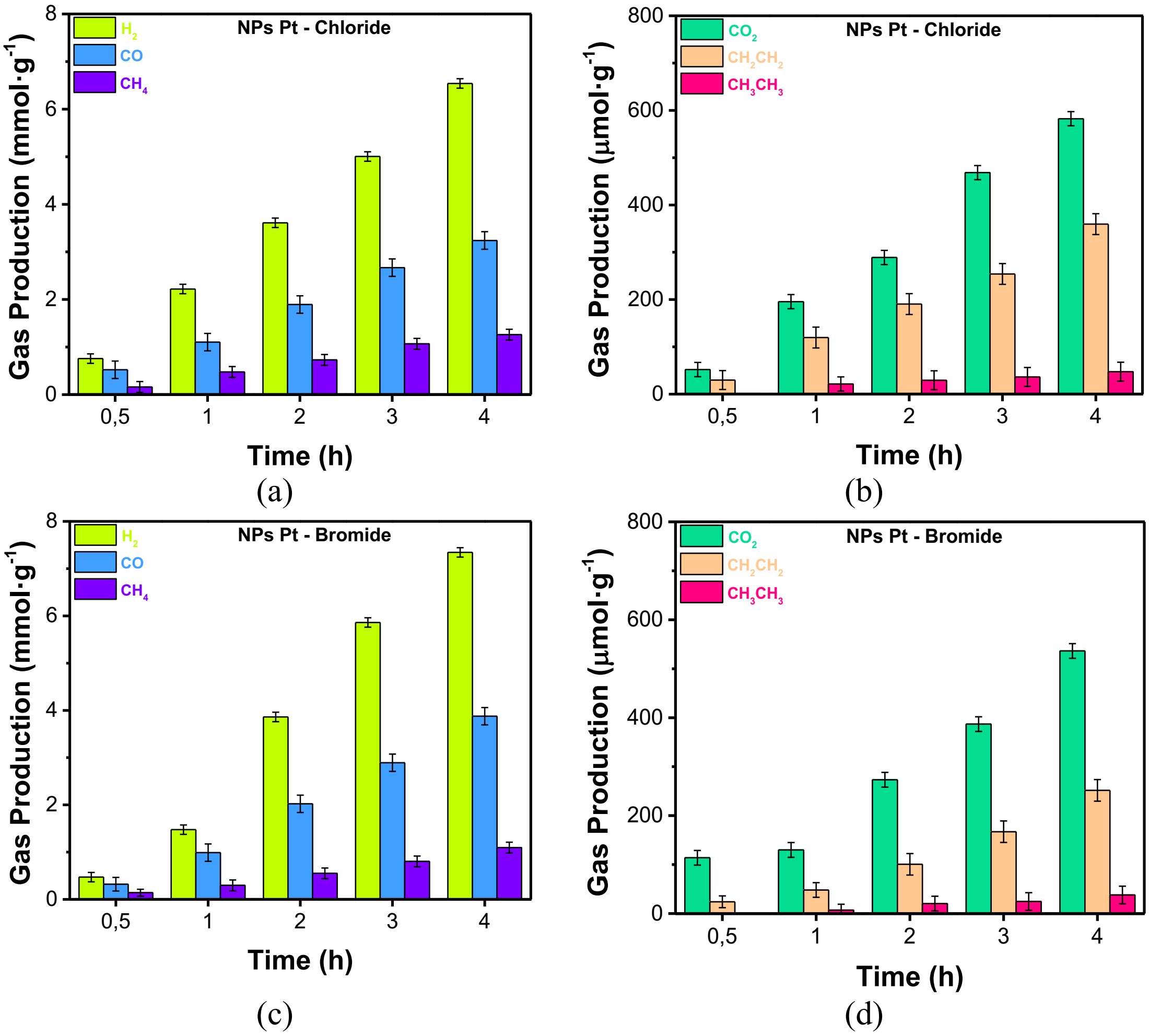

Figure S15. Gas evolution profiles for Pt NPs–chloride: (a) H_2, CO, CH_4 and (b) CO_2, C_2H_4, C_2H_6; and Pt NPs–bromide: (c) H_2, CO, CH_4 and (d) CO_2, C_2H_4, C_2H_6. Dark conditions: Pt NPs (2 mg), P_{Ar} = 1.0 bar, at 4h reaction time and room temperature.

Table S16. Gas-phase products evolved under dark conditions with and without degasification for Pt NPs-chlorine and Pt NPs-bromide catalysts. The table compares gas production (H_2 and hydrocarbons) with and without a 15-minute sonication step to degas the reaction solution, providing insights into sonochemical activity.

Catalyst	Dark Conditions	H_2	CO_2	CH_4	CO	C_2H_4	C_2H_6
		(µmol·g^{-1})					
Pt NPs-chlorine	Without Degas	25.32	97.4	ND	ND	ND	ND
	Degas	5236.8	205.2	593.7	2174.0	137.5	20.3
Pt NPs-bromide	Without Degas	21.5	57.2	ND	ND	ND	ND
	Degas	1467.3	137.8	130.3	588.3	25.2	6.8

ND: No detected

Photocatalytic reaction | Carbon Balance, Selectivity, and Methanol Conversion Calculation

The following equations describe the key parameters used to evaluate the performance of the photocatalytic methanol reforming reaction (MRR). Equation (1) calculates the Carbon Balance (BC), ensuring carbon conservation in both the gas and liquid phases during the reaction. Equation (2) defines Selectivity (S_x), indicating the proportion of each individual product in relation to the total gaseous output. Finally, Equation (3) determines the Methanol conversion (X_{MeOH}), quantifying the amount of methanol converted into products. These equations are essential for assessing the efficiency, selectivity, and conversion rates of the catalytic process, which have been widely used in previous studies of reforming reactions.

Balance Carbon (BC):

$$BC\ (\%) = \frac{n_{C_{liquid\ final}} + n_{C_{gas\ final}}}{n_{C_{liquid\ initial}}} * 100\%$$

$$BC\ (\%) = \frac{\left[(1*CH_4+1*CO+1*CO_2+2*C_2H_4+2*C_2H_6) + n_{C_{liquid\ final}}\right]}{n_{C_{liquid\ initial}}} * 100\% \qquad (1)$$

Selectivity, S_X (%):

$$S_X(\%) = \frac{[X]_{out}}{\left[\sum n_{C_{gas}}\right]_{out}} * 100\% \qquad (2)$$

CH_3OH conversion, X_{MeOH} (%):

$$X_{MeOH}\ (\%) = \frac{\sum n_{C_{gas\ out}}}{n_{MeOH_{in}}} * 100\% \qquad (3)$$

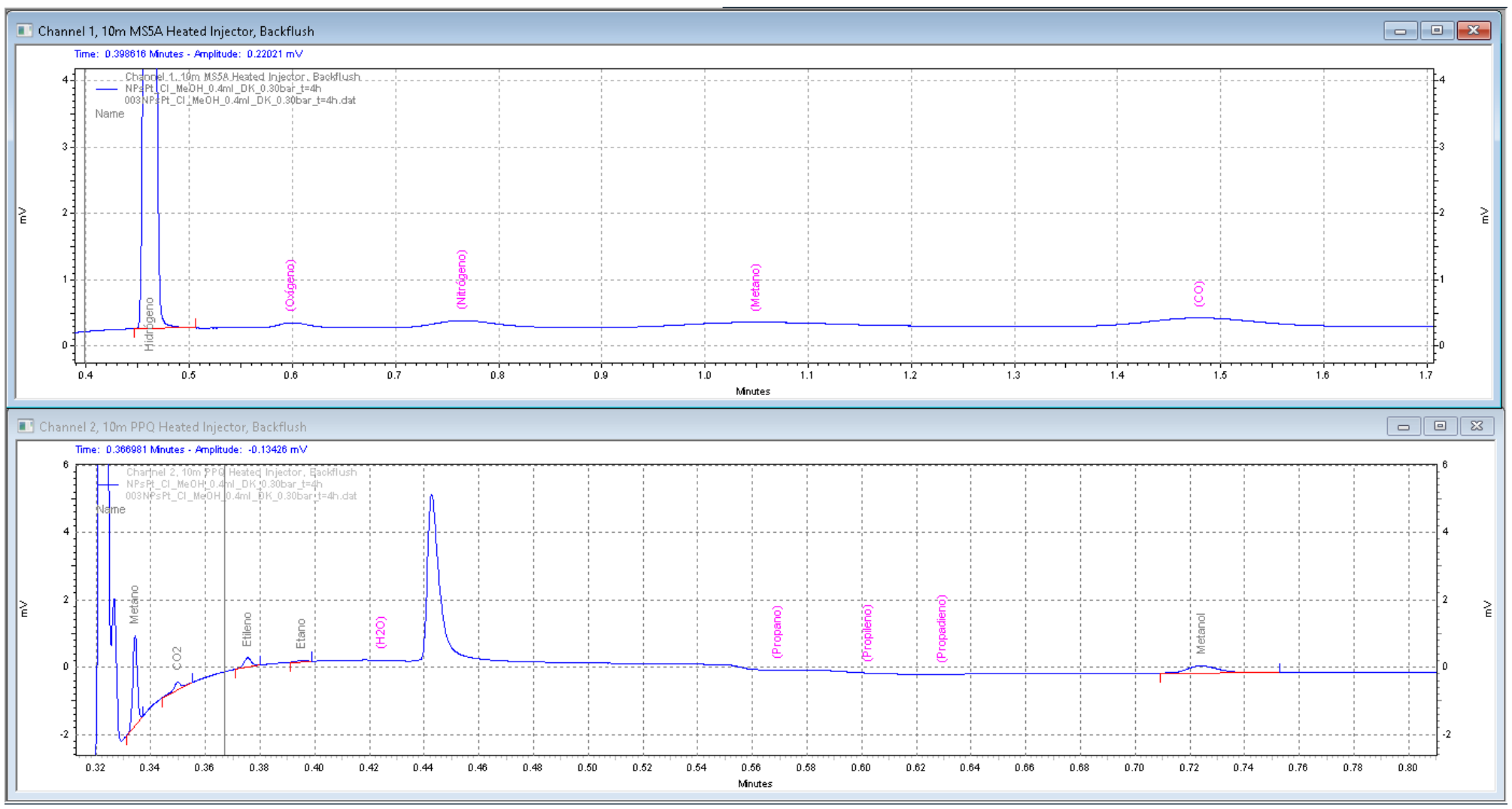

(a)

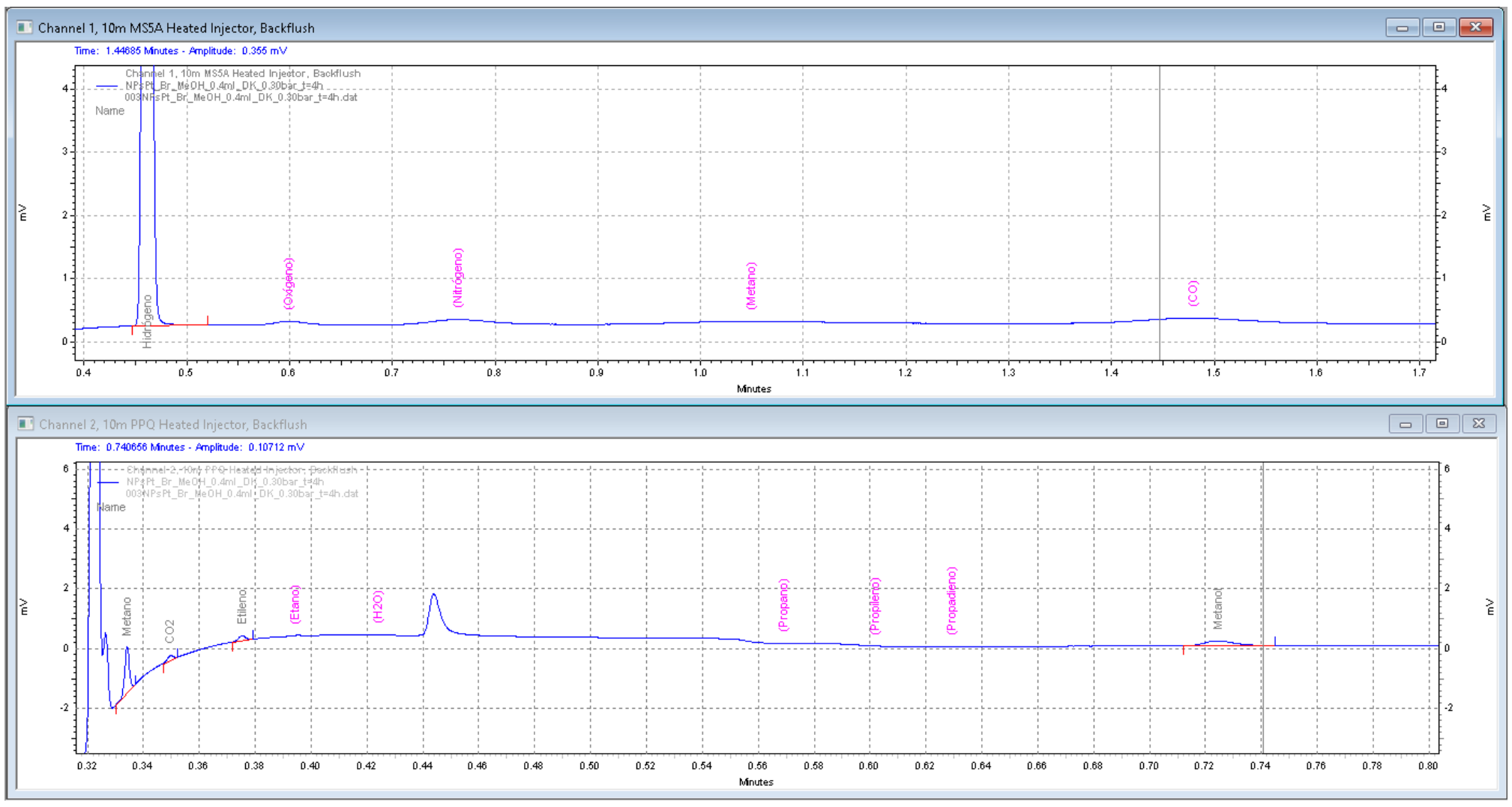

(b)

Figure S17. Representative gas chromatograms (GC) illustrating the reaction product profile obtained during methanol reforming. Both Pt (a) NPs-chlorine and (b) NPs-bromide present the MS5A column output (top) identifying hydrogen and carbon monoxide, and the PPQ column output (bottom) showing methane, carbon dioxide, light hydrocarbons, and gas phase methanol.

Table S18. Catalytic performance and product selectivity for the sunlight-driven methanol reforming reaction.

Catalyst	Conversion X_{MeOH} (%)	S_{CO} (%)	S_{CH_4} (%)	S_{CO_2} (%)	$S_{C_2H_4}$ (%)	$S_{C_2H_6}$ (%)	Carbon Balance (%)
Pt NPs-chlorine	0.28	66.0	15.9	8.2	8.7	1.2	82.4
Pt NPs-bromide	0.24	67.5	14.2	9.5	7.8	1.1	80.5

Table S19. Product selectivity for the dark conditions methanol reforming reaction.

Catalyst	S_{CO} (%)	S_{CH_4} (%)	S_{CO_2} (%)	$S_{C_2H_4}$ (%)	$S_{C_2H_6}$ (%)
Pt NPs-chlorine	61.6	20.8	8.2	8.4	1.1
Pt NPs-bromide	64.8	17.5	9.0	7.6	1.1

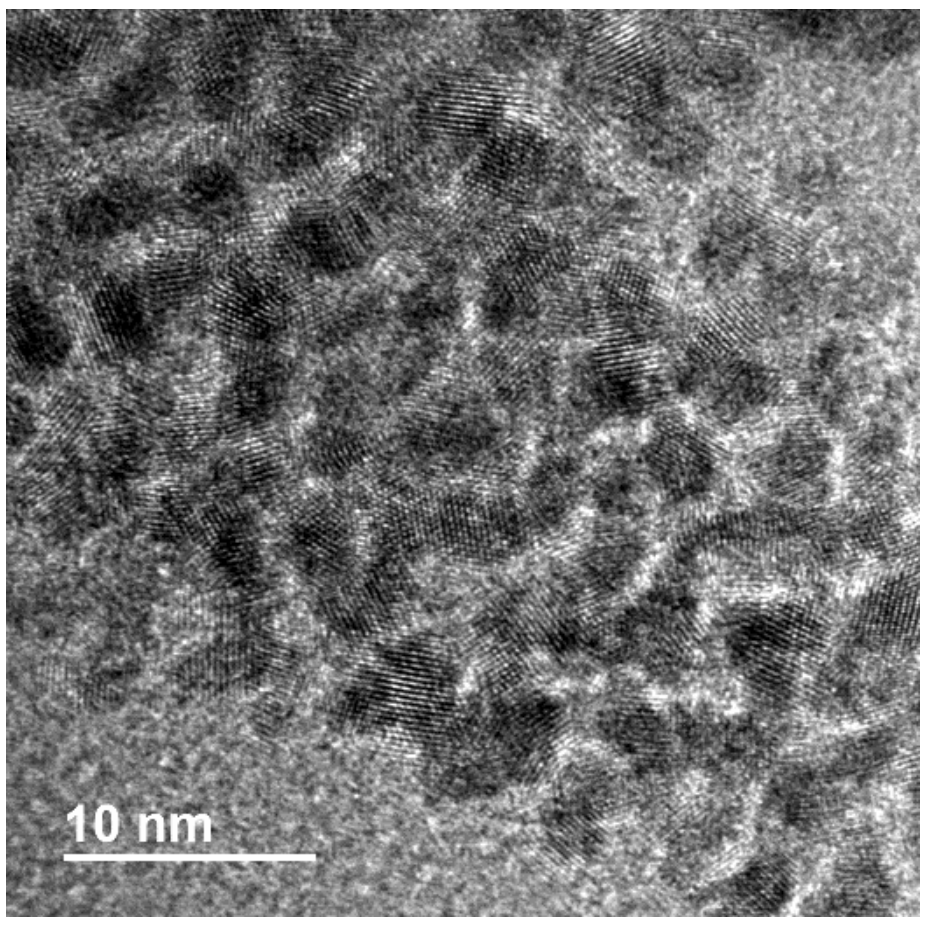

(a)

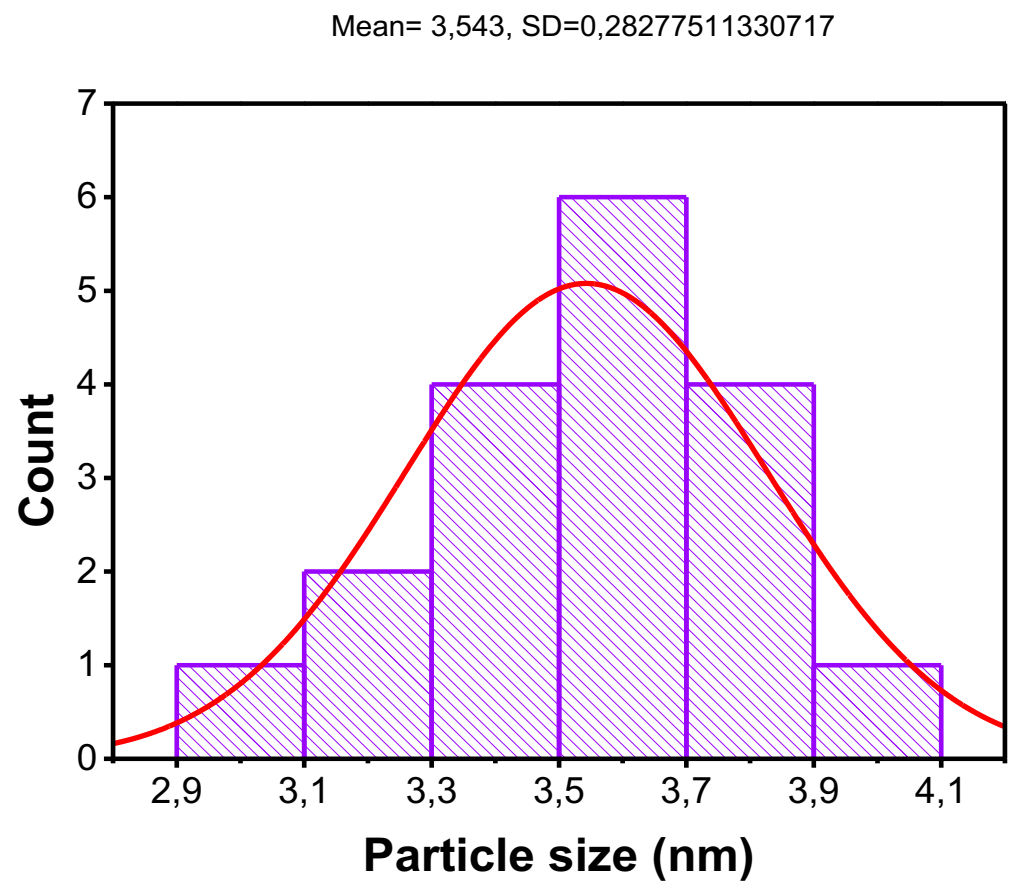

(b)

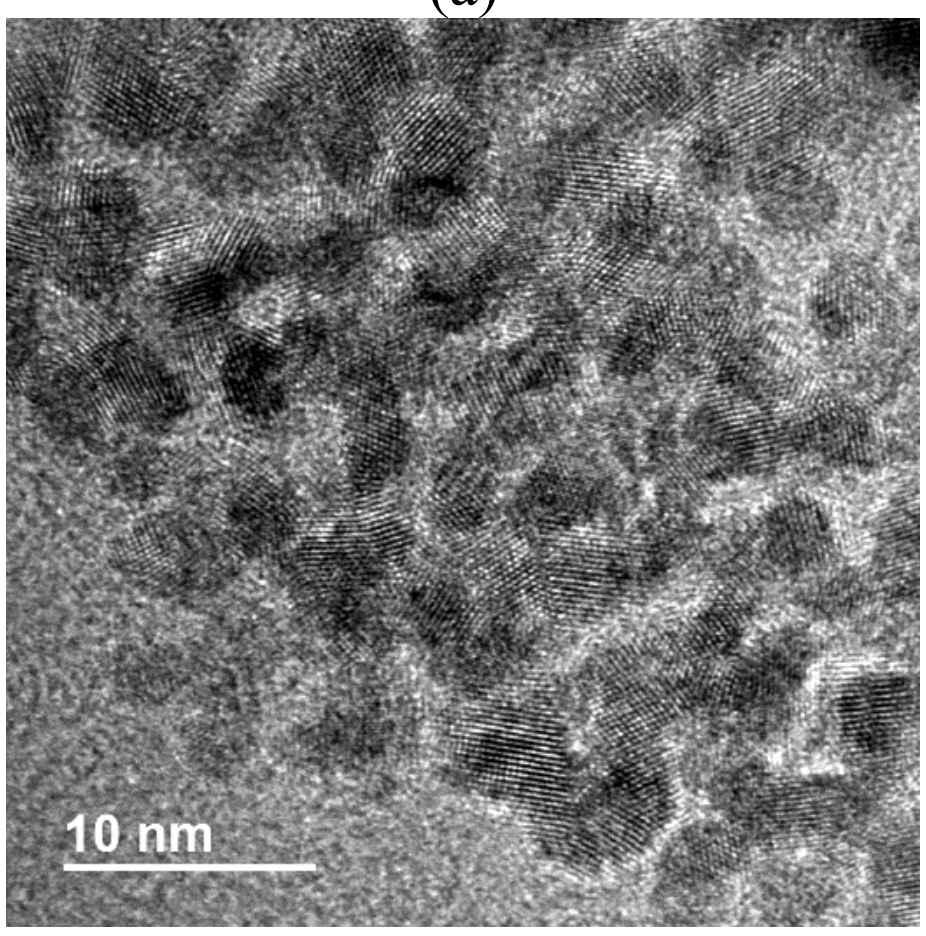

(c)

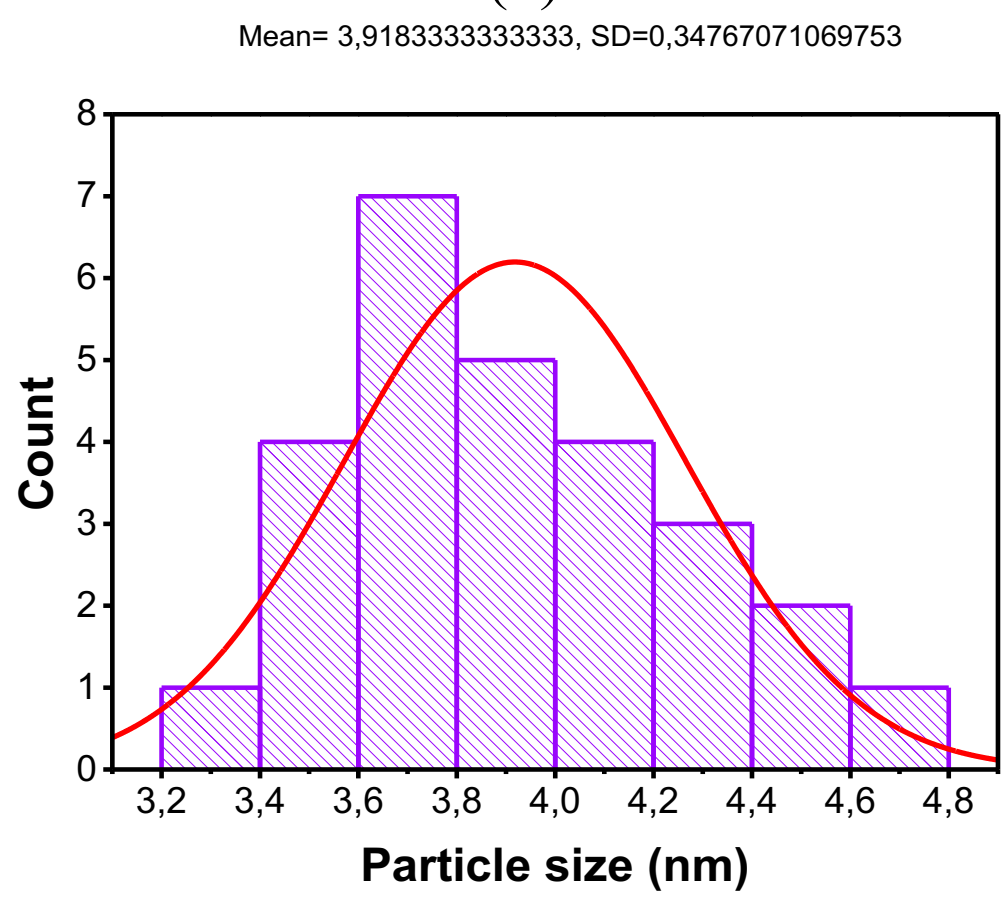

(d)

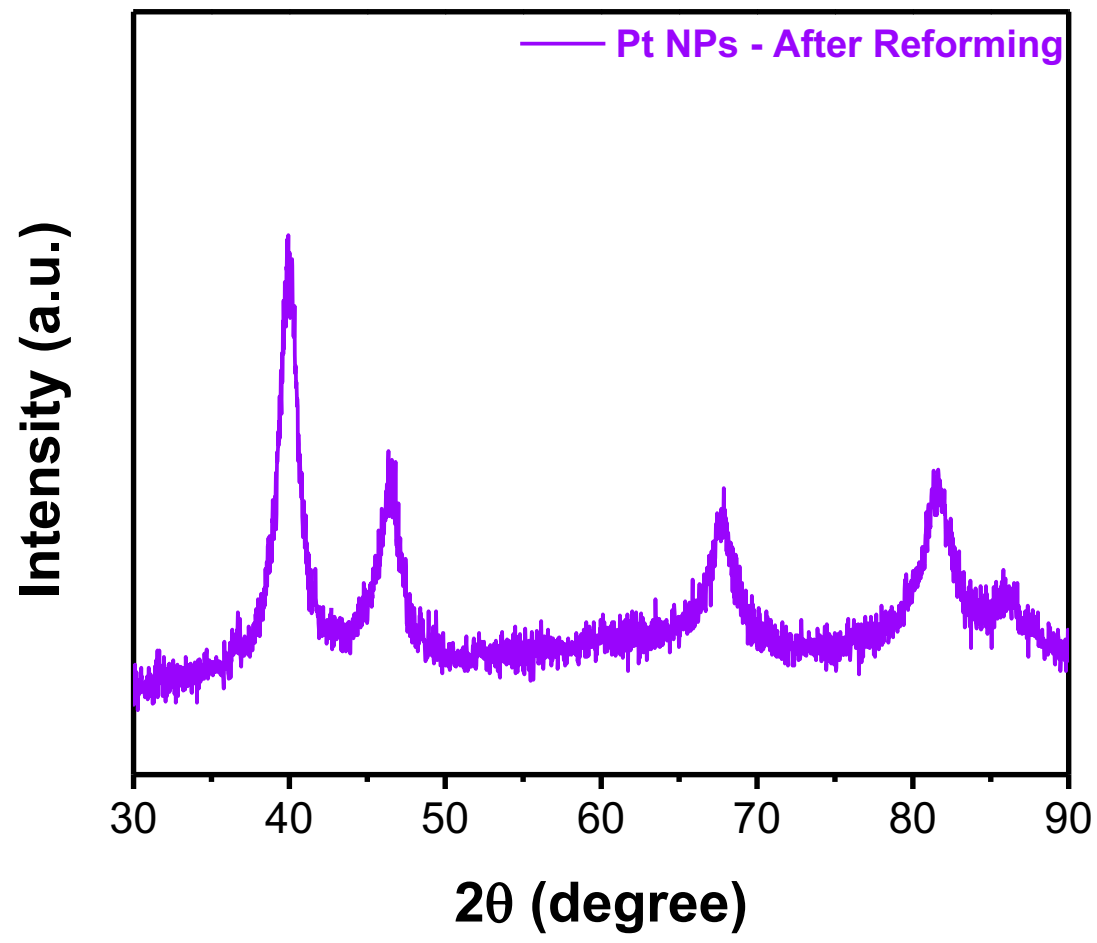

(e)

Figure S20. HR-TEM images and particle size distribution of platinum nanoparticles after methanol reforming reaction for 4 hours under simulated sunlight irradiation. (a, b) Pt NPs-chloride with a mean size of 3.4 nm. (c, d) Pt NPs-bromide with a mean size of 3.8 nm. (e) XRD pattern of Pt NPs after methanol reforming at 4 hours of reaction, showing the crystallinity of the nanoparticles post-catalysis.

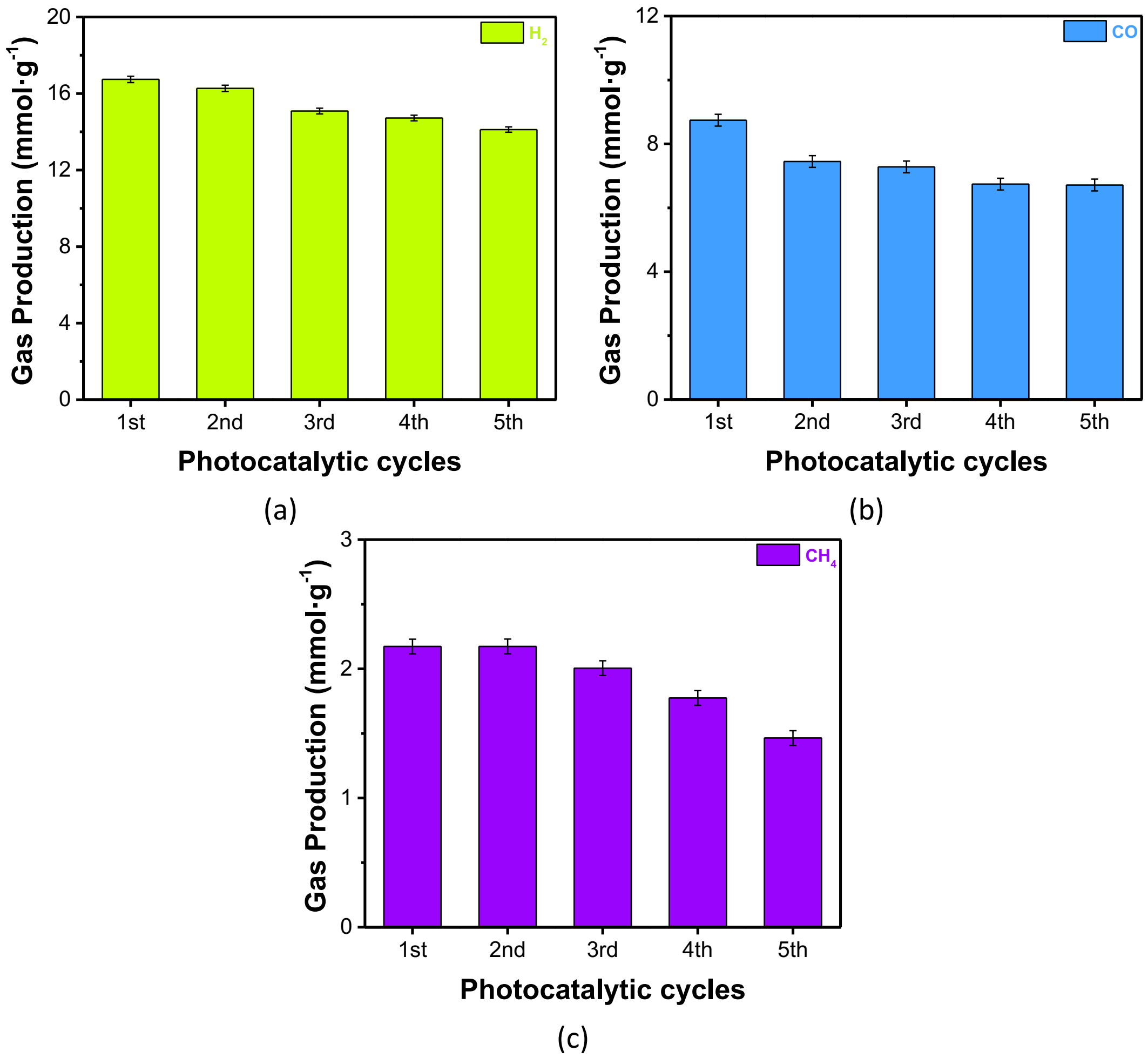

Figure S21. Recycling of the photocatalyst NPs-Pt: evolution of (a) H_2, (b) CO, (c) CH_4. Reaction conditions: photocatalyst (2mg), P_{Ar} = 1.0 bar, at 4h reaction time, simulated sunlight irradiation (130 mW cm^{-2}), and room temperature.

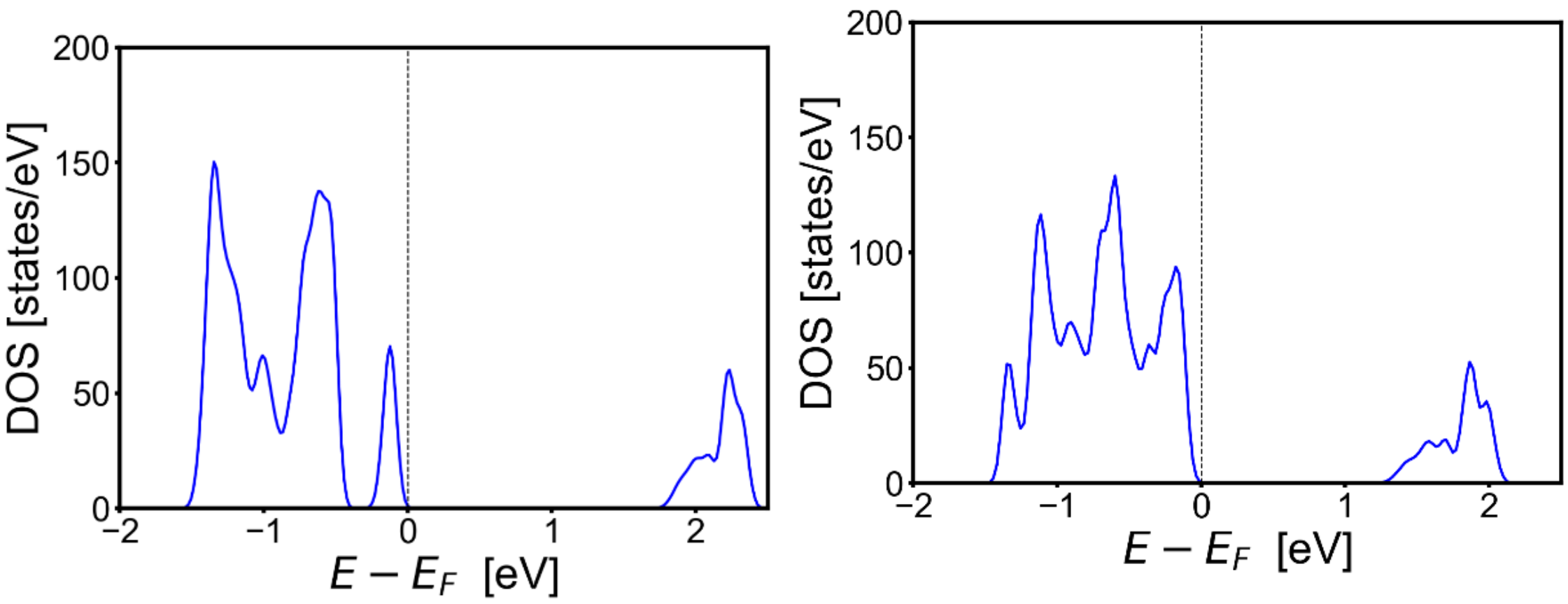

(a) (b)

Figure S22. Total DOS of Cs_2PtX_6 (bulk) computed with PAW–PBE + SOC; energies plotted as E−E_F: (a) Cs_2PtCl_6 and (b) Cs_2PtBr_6. Both are semiconductors with DOS(E_F) = 0; Cs_2PtBr_6 shows a smaller E_g.

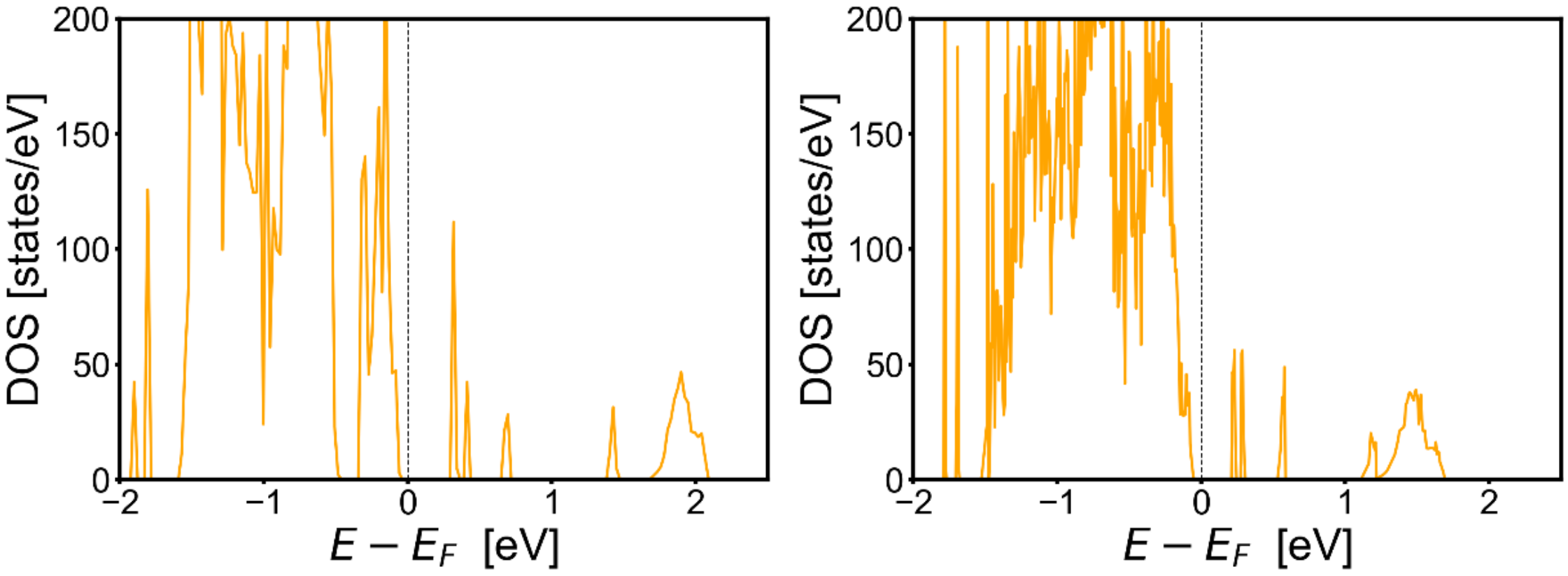

(a) (b)

Figure S23. DOS with a single halide vacancy (V_X); energies plotted as E− E_F : (a) Cs_2PtCl_6 and (b) Cs_2PtBr_6. V_X introduces a narrow in-gap state near E_F with strong Pt-5d character at the Pt adjacent to V_X; in Br this donor lies closer to E_F.

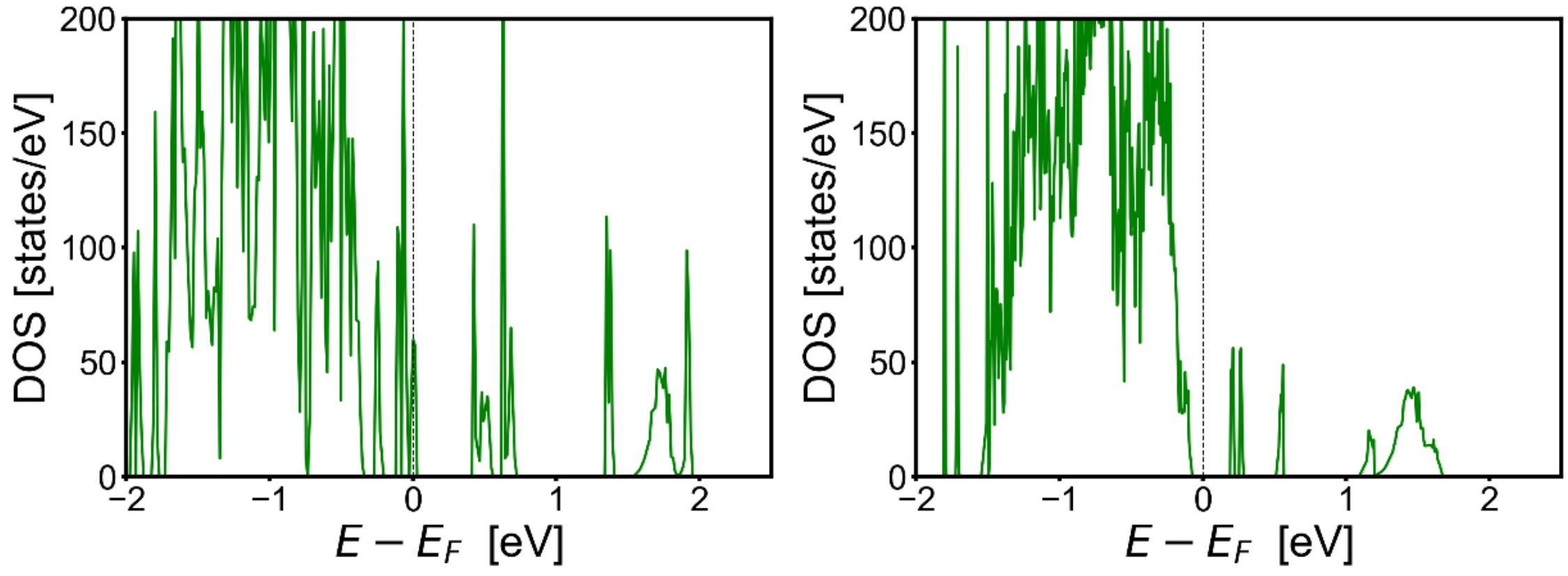

(a) (b)

Figure S24. DOS for $V_X + e^-$ (one excess electron per supercell); energies plotted as $E - E_F$: (a) Cs_2PtCl_6 and (b) Cs_2PtBr_6. The vacancy-centred level becomes occupied, yielding finite DOS at E_F and localized Pt-5d weight.

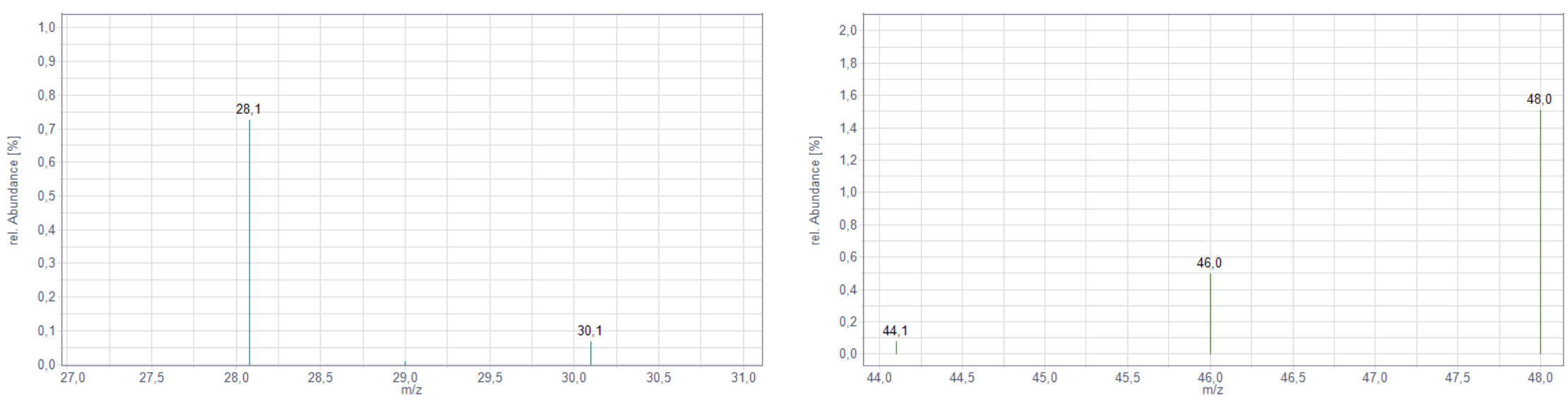

(a) (b)

Figure S25. GC–MS spectra after photocatalytic methanol reforming over Pt NPs under isotope-labelling conditions, using $H_2{}^{18}O$ experiment. Panels show the signals assigned to (a) CO, and (b) CO_2, together with their corresponding $C^{18}O$, $CO^{18}O$ and $C^{18}O_2$ isotopes.

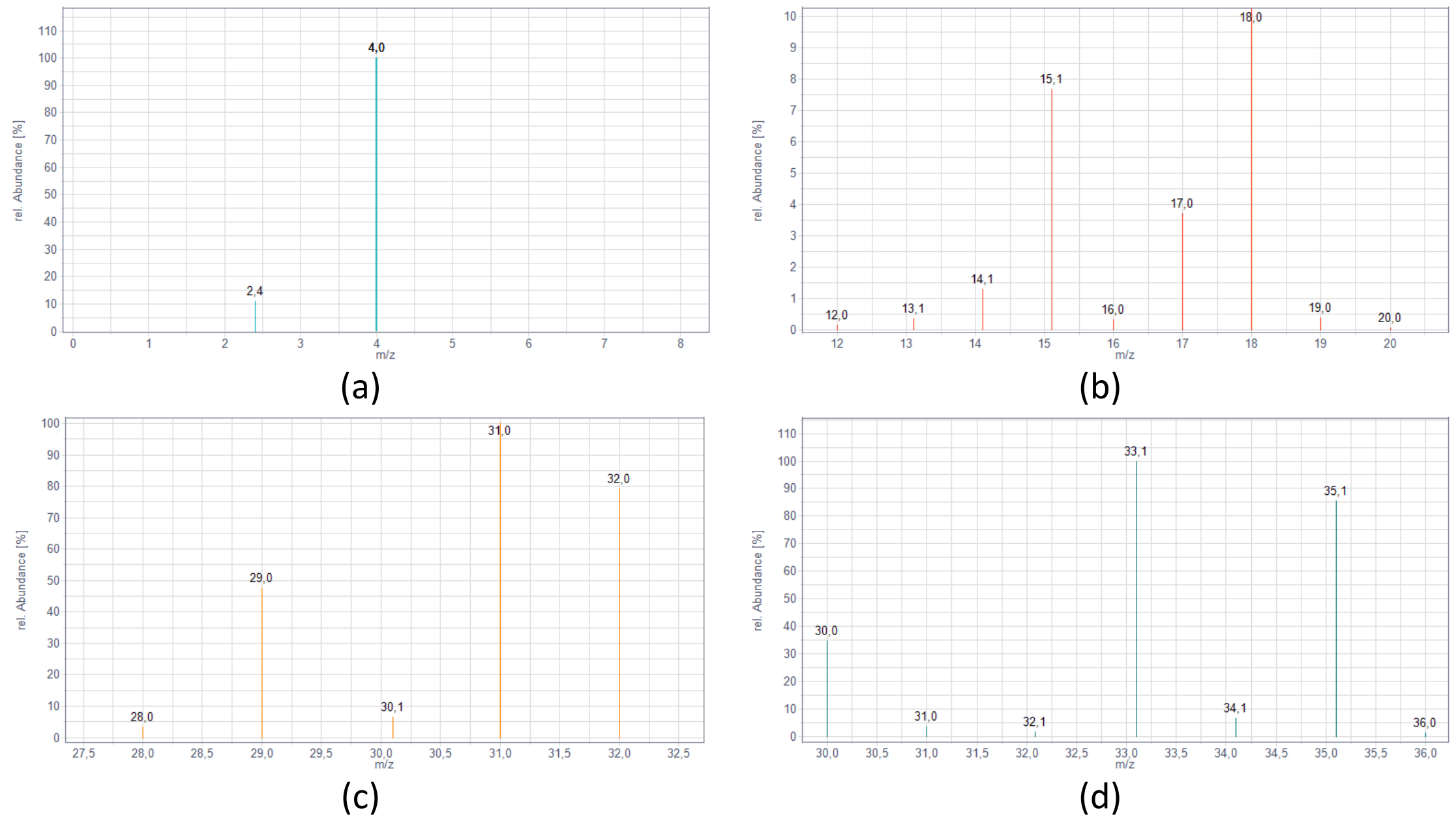

Figure S26. GC–MS spectra of the gaseous products formed after photocatalytic methanol reforming over Pt NPs under isotope-labelling conditions, using D_2O and CD_3OD in separate experiments. Panels show the signals assigned to (a) H_2, (b) CH_4, (c) C_2H_4, and (d) C_2H_6, together with their corresponding deuterated isotopes.

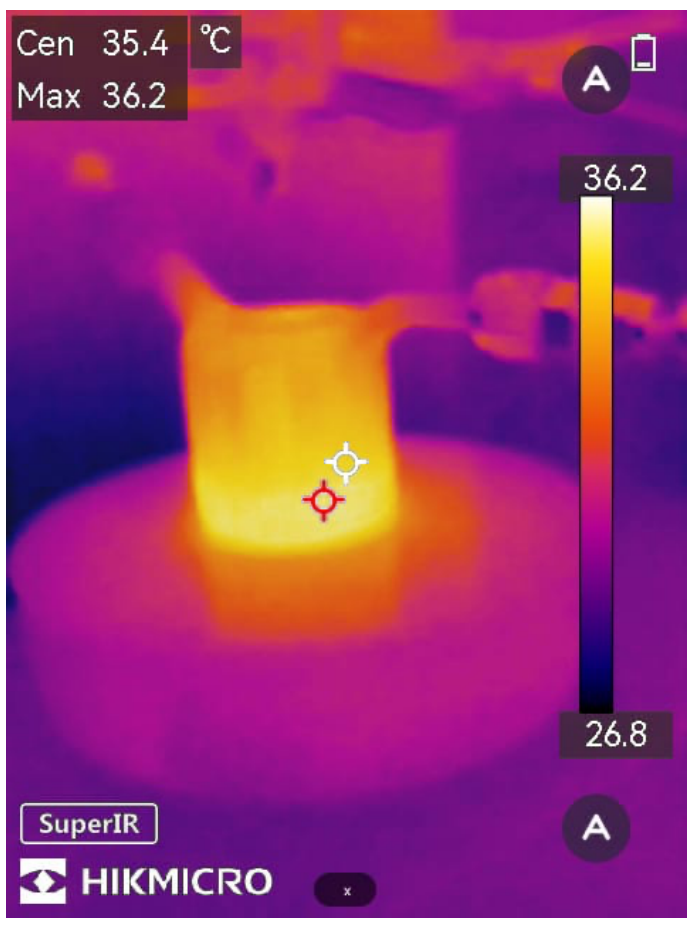

Figure S2728. Surface temperatures of the quartz reactor were monitored using an infrared thermal camera (HIKMICRO SuperIR). The temperature readings ranged from 26.8 °C to 36.2 °C, with the highest measured at the centre of the reactor, providing insights into the thermal distribution during the experiment.

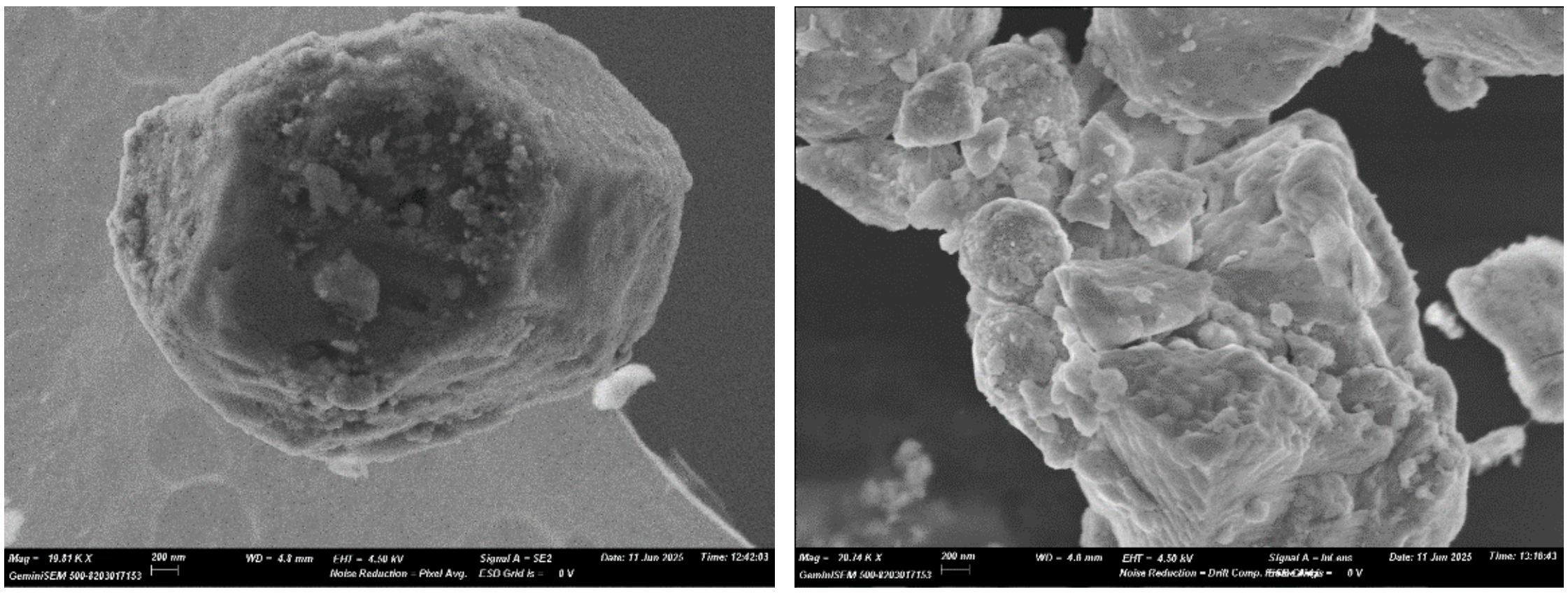

(a) (b)

Figure S2930. FESEM analysis of (a) Cs_2PtCl_6 and (b) Cs_2PtBr_6 under light irradiation during photocatalysis in water. The images show the surface morphology of the perovskites after prolonged light irradiation (t = 23 h). Scale bar: 200 nm. Retrieved from Mego et al., *Adv. Energy Sustain. Res.*, 2025.[12]

References:

(1) Padelkar, S. S.; Vikram; Jasieniak, J. J.; Simonov, A. N.; Alam, A. Mixed-Halide Vacancy-Ordered Double Perovskite for Photovoltaic and Photocatalysis Applications. *Phys. Rev. Appl.* **2024**, *21* (4), 044031. https://doi.org/10.1103/PhysRevApplied.21.044031.

(2) Maughan, A. E.; Ganose, A. M.; Bordelon, M. M.; Miller, E. M.; Scanlon, D. O.; Neilson, J. R. Defect Tolerance to Intolerance in the Vacancy-Ordered Double Perovskite Semiconductors Cs2SnI6 and Cs2TeI6. *J. Am. Chem. Soc.* **2016**, *138* (27), 8453–8464. https://doi.org/10.1021/JACS.6B03207/SUPPL_FILE/JA6B03207_SI_008.CIF.

(3) Wojnicki, M.; Kwolek, P. Reduction of Hexachloroplatinate(IV) Ions with Methanol under UV Radiation. *J. Photochem. Photobiol. A Chem.* **2016**, *314*, 133–142. https://doi.org/10.1016/j.jphotochem.2015.08.020.

(4) Harada, M.; Einaga, H. Formation Mechanism of Pt Particles by Photoreduction of Pt Ions in Polymer Solutions. *Langmuir* **2006**, *22* (5), 2371–2377. https://doi.org/10.1021/la052378m.

(5) Escobar Morales, B.; Gamboa, S. A.; Pal, U.; Guardián, R.; Acosta, D.; Magaña, C.; Mathew, X. Synthesis and Characterization of Colloidal Platinum Nanoparticles for Electrochemical Applications. *Int. J. Hydrogen Energy* **2010**, *35* (9), 4215–4221. https://doi.org/10.1016/j.ijhydene.2010.01.040.

(6) Karthik, R.; Sasikumar, R.; Chen, S. M.; Govindasamy, M.; Vinoth Kumar, J.; Muthuraj, V. Green Synthesis of Platinum Nanoparticles Using Quercus Glauca Extract and Its Electrochemical Oxidation of Hydrazine in Water Samples. *Int. J. Electrochem. Sci.* **2016**, *11* (10), 8245–8255. https://doi.org/10.20964/2016.10.62.

(7) Eramabadi, P.; Masoudi, M.; Makhdoumi, A.; Mashreghi, M. Microbial Cell Lysate Supernatant (CLS) Alteration Impact on Platinum Nanoparticles Fabrication, Characterization, Antioxidant and Antibacterial Activity. *Mater. Sci. Eng. C* **2020**, *117*, 111292. https://doi.org/10.1016/j.msec.2020.111292.

(8) Jameel, M. S.; Aziz, A. A.; Dheyab, M. A. Comparative Analysis of Platinum Nanoparticles Synthesized Using Sonochemical-Assisted and Conventional Green Methods. *Nano-Structures & Nano-Objects* **2020**, *23*, 100484. https://doi.org/10.1016/j.nanoso.2020.100484.

(9) Ji, W.; Qi, W.; Tang, S.; Peng, H.; Li, S. Hydrothermal Synthesis of Ultrasmall Pt Nanoparticles as Highly Active Electrocatalysts for Methanol Oxidation. *Nanomater. 2015, Vol. 5, Pages 2203-2211* **2015**, *5* (4), 2203–2211. https://doi.org/10.3390/NANO5042203.

(10) Lazar, O. A.; Nikolov, A. S.; Anicai, L.; Mihai, G. V.; Messina, A. A.; Enachescu, M. Synthesis of Platinum Nanoparticles by Pulsed Laser Ablation with an Excimer KrF Laser in Deep Eutectic Solvents. *ACS Omega* **2025**, *10* (8), 8066–8081. https://doi.org/10.1021/acsomega.4c09164.

(11) Martínez-Rodríguez, R. A.; Vidal-Iglesias, F. J.; Solla-Gullón, J.; Cabrera, C. R.; Feliu, J. M. Synthesis of Pt Nanoparticles in Water-in-Oil Microemulsion: Effect of HCl on Their Surface Structure. *J. Am. Chem. Soc.* **2014**, *136* (4), 1280–1283. https://doi.org/10.1021/ja411939d.

(12) Mego, K.; Ruiz-Campos, P.; Baldoví, H. G.; Atienzar, P. OER Activity Promoted by Organic Ligand-Free Cs2Pt(Cl, Br)6 Perovskite Photocatalyst for Solar-Driven Water Splitting. *Adv. Energy Sustain. Res.* **2025**, 2500105. https://doi.org/10.1002/aesr.202500105.
